# Antibacterial activity of novel dual bacterial DNA type II topoisomerase inhibitors

**DOI:** 10.1371/journal.pone.0228509

**Published:** 2020-02-19

**Authors:** Noemi D’Atanasio, Alessandra Capezzone de Joannon, Laura Di Sante, Giorgina Mangano, Rosella Ombrato, Marco Vitiello, Cristina Bartella, Gabriele Magarò, Federica Prati, Claudio Milanese, Carla Vignaroli, Francesco Paolo Di Giorgio, Serena Tongiani

**Affiliations:** 1 Angelini RR&D (Regulatory, Research & Development)–Angelini S.p.A., S. Palomba-Pomezia (Rome), Italy; 2 Department of Life and Environmental Sciences, Polytechnic University of Marche, Ancona, Italy; University of Nebraska Medical Center, UNITED STATES

## Abstract

In this study, a drug discovery programme that sought to identify novel dual bacterial topoisomerase II inhibitors (NBTIs) led to the selection of six optimized compounds. In enzymatic assays, the molecules showed equivalent dual-targeting activity against the DNA gyrase and topoisomerase IV enzymes of *Staphylococcus aureus* and *Escherichia coli*. Consistently, the compounds demonstrated potent activity in susceptibility tests against various Gram*-*positive and Gram-negative reference species, including ciprofloxacin-resistant strains. The activity of the compounds against clinical multidrug-resistant isolates of *S*. *aureus*, *Clostridium difficile*, *Acinetobacter baumannii*, *Neisseria gonorrhoeae*, *E*. *coli* and vancomycin-resistant *Enterococcus* spp. was also confirmed. Two compounds (**1** and **2**) were tested in time-kill and post-antibiotic effect (PAE) assays. Compound **1** was bactericidal against all tested reference strains and showed higher activity than ciprofloxacin, and compound **2** showed a prolonged PAE, even against the ciprofloxacin-resistant *S*. *aureus* BAA-1720 strain. Spontaneous development of resistance to both compounds was selected for in *S*. *aureus* at frequencies comparable to those obtained for quinolones and other NBTIs. *S*. *aureus* BAA-1720 mutants resistant to compounds **1** and **2** had single point mutations in *gyrA* or *gyrB* outside of the quinolone resistance-determining region (QRDR), confirming the distinct site of action of these NBTIs compared to that of quinolones. Overall, the very good antibacterial activity of the compounds and their optimizable *in vitro* safety and physicochemical profile may have relevant implications for the development of new broad-spectrum antibiotics.

## Introduction

The need for new antibiotics that possess innovative mechanisms of action and are able to overcome antibacterial resistance to currently available drugs is recognized worldwide. The emergence of resistance to multiple antibacterial agents in pathogenic bacteria is becoming a significant threat to public health [1]. Moreover, the frequent recovery of clinical isolates simultaneously resistant to most antibiotic classes, including last-line antibacterials, such as colistin [2], is a clear indicator of the extent and impact of the worldwide problem of antibacterial resistance [3].

Bacterial DNA gyrase and topoisomerase IV (topo IV) are “highly conserved” type II topoisomerases that play essential roles in promoting DNA replication and transcription [4, 5]. The mechanism of action of these enzymes, which are essential in controlling bacterial DNA topology, involves DNA cleavage and the passage of a second DNA double strand through the break, followed by re-ligation of the cleaved DNA [6, 7]. Both DNA gyrase and topo IV are highly homologous functional heterotetramers whose subunits are referred to as GyrA/GyrB and ParC/ParE, respectively. The GyrB and ParE subunits include an ATPase domain that, by catalysing ATP hydrolysis, provides the energy necessary for the enzymatic-induced cleavage, which takes place under the control of the other subunits [8]. DNA gyrase acts by introducing negative supercoils into the DNA molecule and is involved in DNA elongation, whereas the action of topo IV consists of decatenation of daughter chromosomes and DNA relaxation [9]. Both supercoiling and decatenation are essential control mechanisms during cellular replication, and compounds interfering with these reactions ultimately cause the death of bacterial cells [10].

Topoisomerase II enzymes are validated bacterial targets [10, 11] for two classes of antibiotic drugs, the aminocoumarins, such as novobiocin, which inhibit the ATPase domain of the enzymes [12] but have limited clinical usage due to their toxicity [13], and the fluoroquinolones. The latter are one of the most successful classes of antibiotics and are widely used to treat a large variety of Gram-positive and Gram-negative bacterial infections [14]. However, the U.S. regulatory authorities have recently imposed severe limitations on their use as first-line drugs in a number of acute infections due to their side effects [15]. Fluoroquinolones act by binding to the GyrA and ParC subunits of DNA gyrase and topo IV, forming a ternary drug-enzyme-DNA complex that causes breaks in double-stranded DNA and leads to blockage of DNA replication and transcription [16, 17]. Despite the dual-target mechanism of action of fluoroquinolones, the incidence of resistance to these drugs has increased significantly in the last two decades in both Gram-positive and Gram-negative bacteria [18]. To overcome increasing resistance to fluoroquinolones, a class of novel bacterial topoisomerase inhibitors (NBTIs) that are structurally different from quinolones and use a distinct mechanism to trap the topoisomerase-DNA complex was described in literature [19]. Structurally, NBTI molecules comprise left hand side (LHS) group that interacts with the DNA, a central linker portion, and a right hand side (RHS) group that binds to the enzymes [20]. A number of advanced NBTI molecules have been described in the literature, including NXL101 [21], AZD9742 [22], NBTI 5463 [23] and gepotidacin [24]. Both quinolones and NBTIs interact with the protein-DNA complex, but while quinolones first bind to the enzymes via interactions between their catalytic tyrosines and the broken double-stranded DNA, NBTIs bind to the enzymes when the DNA is still unbroken [19]. Due to their novel mechanism of action, NBTIs have the potential to become a new class of therapeutic agents for the treatment of infectious diseases.

**Fig 1:**
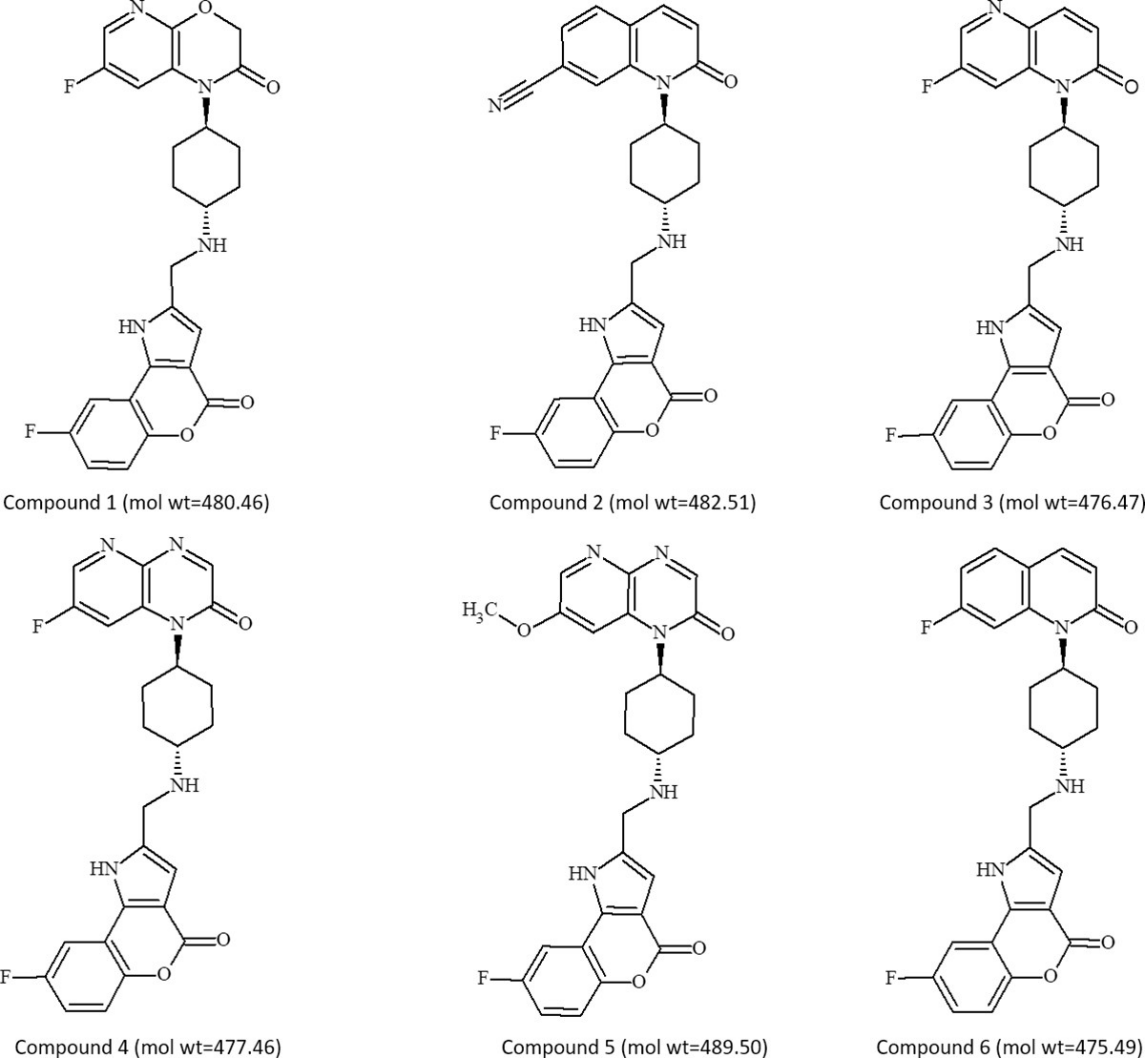
Chemical structures and molecular weights of optimized NBTI hit compounds.

On these premises, we embarked on a drug discovery program aimed at identifying novel NBTIs. Computational screening approaches and subsequent optimization strategies were carried out, leading to the discovery of an original N-linked piperazine series with dual activity against DNA gyrase and topo IV from *E*. *coli* and *S*. *aureus* and promising antibacterial activity [25]. Further medicinal and computational chemistry efforts provided a novel benzopyrano[4,3]pyrrol-4(1H)-one class. Herein, representative compounds **1**–**6** (Fig 1) are investigated for the antibacterial effects against Gram-positive and Gram-negative strains and clinically relevant resistant isolates.

## Materials and methods

### Synthesis

Compounds **1**–**6** (Fig 1) were synthesized in the Angelini chemistry laboratory via a previously reported synthetic route [26] based on i) preparation of the common tricyclic system 8-fluoro-4-oxo-1,4-dihydro[1]benzopyrano[4,3-b]pyrrole-2-carbaldehyde and ii) subsequent coupling with the appropriate 4-substituted-cyclohexylamines (see details in S1 File).

8-Fluoro-2-({[trans-4-(7-fluoro-2-oxo-2,3-dihydro-1H-pyrido[2,3-b][1,4]oxazin-1-yl)cyclohexyl]amino}methyl)[1]benzopyrano[4,3-b]pyrrol-4(1H)-one (Compound **1**). LC-MS (ESI) m/z: 481.3 [M+H]^+^. HRMS (ESI): calculated 481.1682 [M+H]^+^, found 481.1697 [M+H]^+^. ^1^H-NMR (300 MHz, DMSO-*d*_*6*_) δ: 1.15–1.41 (m, 2 H), 1.72 (d, J = 10.74 Hz, 2 H), 2.01 (d, J = 1206 Hz, 2 H), 2.26–2.38 (m, 2 H), 2.48–2.56 (m, 1H), 3.30 (br s, 1 H), 3.87 (s, 2H), 4.01–4.10 (m, 1 H), 4.69 (s, 2 H), 6.58 (s, 1 H), 7.28 (td, J = 8.75, 3.06 Hz, 1 H), 7.47 (dd, J = 9.17, 4.62 Hz, 1 H), 7.82–7.88 (m, 2 H), 7.94 (dd, J = 9.12, 3.01 Hz, 1 H), 12.44 (br s, 1 H). ^13^C-NMR (75 MHz, DMSO-*d*_*6*_) δ: 26.6, 31.9, 43.0, 54.7, 56.1, 67.5, 104.7, 106.9, 108.4, 112.8, 114.8, 115.2, 118.7, 125.5, 126.5, 134.0, 138.9, 147.3, 148.6, 155.8, 157.9, 158.0, 165.0.

1-(Trans-4-{[(8-fluoro-4-oxo-1,4-dihydro[1]benzopyrano[4,3-b]pyrrol-2-yl)methyl]amino} cyclohexyl)-2-oxo-1,2-dihydroquinoline-7-carbonitrile hydrochloride (Compound **2**). LC-MS (ESI) m/z: 483.3 [M+H]^+^. HRMS (ESI): calculated 483.1827 [M+H]^+^, found 483.1825 [M+H]^+^.^1^H-NMR (300 MHz, DMSO-*d*_*6*_) δ: 1.36–1.47 (m, 2H), 1.63 (d, J = 10.54 Hz, 2H), 2.04 (d, J = 11.51 Hz, 2H), 2.52–2.78 (m, 3H), 3.32 (br s, 2H), 3.90 (s, 2H), 4.70 (br s, 1H), 6.59 (s, 1H), 6.67 (d, J = 9.34 Hz, 1H), 7.29 (td, J = 8.77, 3.06 Hz, 1H), 7.47 (dd, J = 9.13, 4.63 Hz, 1H), 7.63 (dd, J = 7.97, 1.05 Hz, 1H), 7.88 (d, J = 7.97 Hz, 1H), 7.90–8.01 (m, 2H), 8.34 (br s, 1H), 12.52 (s, 1H). ^13^C-NMR (75 MHz, DMSO-d_*6*_) δ: 25.4, 27.9, 40.2, 55.1, 107.3, 108.6, 108.8, 112.7, 114.5, 116.0, 118.7, 118.8, 118.9, 124.2, 124.7, 125.4, 129.3, 130.2, 134.9, 138.5, 139.3, 147.8, 157.6, 158.1, 161.6.

8-Fluoro-2-({[trans-4-(7-fluoro-2-oxo-1,5-naphthyridin-1(2H)-yl)cyclohexyl]amino} methyl)[1]benzopyrano[4,3-b]pyrrol-4(1H)-one hydrochloride (Compound **3**). LC-MS (ESI) m/z: 477.2 [M+H]^+^. HRMS (ESI): calculated 478.1734 [M+H]^+^, found 477.1749 [M+H]^+^. ^1^H-NMR (30 MHz, DMSO-*d*_*6*_) δ: 1.64–1.95 (m, 4H), 2.33 (br d, J = 8.75 Hz, 2H), 2.54–2.80 (m, 2H), 3.30 (br s, 1H), 4.42 (br t, J = 4.75 Hz, 2H), 4.71 (br s, 1H), 6.73 (d, J = 9.66 Hz, 1H), 6.96 (d, J = 1.90 Hz, 1H), 7.32 (td, J = 8.79, 3.06 Hz, 1H), 7.48 (dd, J = 9.12, 4.67 Hz, 1H), 7.79–7.95 (m, 2H), 8.31 (dd, J = 11.89, 2.15 Hz, 1H), 8.51 (d, J = 2.15 Hz, 1H), 9.59 (br d, J = 3.80 Hz, 2H), 13.67 (br s, 1H). ^13^C-NMR (75 MHz, DMSO-*d*_*6*_) δ: 25.1, 27.6, 54.9, 106.9, 108.4, 108.6, 109.3, 114.2, 115.8, 118.7, 124.9, 128.7, 132.5, 134.7, 135.0, 136.9, 139.5, 147.6, 157.3, 157.8, 158.7, 161.1

8-fluoro-2-({[*trans*-4-(7-fluoro-2-oxopyrido[2,3-b]pyrazin-1(2H)-yl)cyclohexyl]amino}methyl)[1]benzopyrano[4,3-b]pyrrol-4(1H)-one formate salt (Compound **4**). LC-MS (ESI) m/z: 478.4 [M+H]^+^. HRMS (ESI): calculated 478.1685 [M+H]^+^, found 478.1700 [M+H]^+^. ^1^H-NMR (400 MHz, DMSO-*d*_*6*_) δ: 1.41 (m, J = 11.80 Hz, 2 H), 1.70 (d, J = 11.29 Hz, 2 H), 2.06 (d, J = 10.29 Hz, 2 H), 2.51–2.58 (m, 2 H), 2.67 (br s, 1 H), 3.96 (s, 2 H), 4.47–4.79 (m, 1 H), 6.63 (s, 1 H), 7.30 (td, J = 8.78, 3.01 Hz, 1 H), 7.48 (dd, J = 9.03, 4.52 Hz, 1 H), 7.95 (dd, J = 9.03, 3.01 Hz, 1 H), 8.21 (s, 1 H), 8.29 (s, 1 H), 8.45 (d, J = 11.04 Hz, 1 H), 8.58 (d, J = 2.26 Hz, 1 H). ^13^C-NMR (free base) (75 MHz, DMSO-*d*_*6*_) δ: 25.6, 31.3, 42.8, 54.8, 60.0, 105.0, 107.0, 108.4, 110.8, 114.8, 115.2, 118.7, 130.5, 132.9, 134.2, 138.2, 140.7, 147.4, 154.3, 157.8, 157.9, 160.6, 172.6.

8-Fluoro-2-({[trans-4-(7-methoxy-2-oxopyrido[2,3-b]pyrazin-1(2H)-yl)cyclohexyl]amino}methyl)[1]benzopyrano[4,3-b]pyrrol-4(1H)-one hydrochloride (Compound **5**). LC-MS (ESI) m/z: 490.1 [M+H]^+^. HRMS (ESI): calculated 490.1885 [M+H]^+^, found 490.1888 [M+H]^+^. ^1^H-NMR (500 MHz, DMSO-*d*_*6*_) δ: 1.69–1.79 (m, 2 H), 1.84 (d, J = 10.98 Hz, 2 H), 2.25–2.34 (m, 2 H), 2.56–2.71 (m, 2 H), 3.17–3.35 (m, 1 H), 4.03 (s, 3 H), 4.37–4.45 (m, 2 H), 4.67 (br s, 1 H), 6.96 (d, J = 1.65 Hz, 1 H), 7.38 (td, J = 8.78, 3.02 Hz, 1 H), 7.53 (dd, J = 9.19, 4.53 Hz, 1 H), 7.69–7.81 (m, 1 H), 7.88 (dd, J = 8.65, 2.88 Hz, 1 H), 8.16 (s, 1 H), 8.35 (d, J = 2.47 Hz, 1 H), 9.42 (br s, 2 H), 13.54 (br s, 1 H). ^13^C-NMR (75 MHz, DMSO-d_*6*_) δ: 21.7, 25.7, 31.5, 43.0, 54.9, 56.5, 104.8, 106.3, 107.0, 108.4, 114.9, 115.2, 118.7, 130.2, 133.9, 134.1, 138.4, 138.8, 147.4, 150.9, 154.7, 158.0, 158.1, 172.5.

8-Fluoro-2-({[trans-4-(7-fluoro-2-oxoquinolin-1(2H)-yl)cyclohexyl]amino}methyl)[1] benzopyrano[4,3-b]pyrrol-4(1H)-one hydrochloride (Compound **6**). LC-MS (ESI) m/z: 476.1 [M+H]^+^. HRMS (ESI): calculated 476.1780 [M+H]^+^, found 476.1779 [M+H]^+^. ^1^H-NMR (300 MHz, DMSO-*d*_*6*_) δ: 1.53–1.73 (m, 4 H), 2.16–2.21 (m 2 H), 2.66–2.73 (m, 2 H), 2.80–3.13 (m, 1 H), 4.21 (br s, 2 H), 4.32–4.65 (m, 1 H), 6.47 (d, J = 9.15 Hz, 1 H), 6.81 (s, 1 H), 7.13 (td, J = 8.44, 2.20 Hz, 1 H), 7.34 (td, J = 8.56, 2.93 Hz, 1 H), 7.51 (dd, J = 9.05, 4.65 Hz, 1 H), 7.66–7.81 (m, 2 H), 7.86 (d, J = 9.51 Hz, 1 H), 7.91 (dd, J = 8.80, 2.93 Hz, 1 H), 13.13 (br s, 1 H).

### Antibacterial agents and reagents

Ciprofloxacin hydrochloride was purchased from MP Biomedicals (Santa Ana, CA, USA); erythromycin hydrochloride, vancomycin hydrochloride, ampicillin, ceftriaxone sodium, levofloxacin, imipenem monohydrate, gentamicin sulfate, tetracycline hydrochloride, colistin sulfate, penicillin G potassium, teicoplanin, linezolid, clindamycin hydrochloride, and metronidazole were purchased from Sigma-Aldrich (St Louis, MO, USA); meropenem was purchased from TCI (Portland, OR, USA); and daptomycin and fidaxomicin were purchased from Selleckchem (Houston, TX, USA).

### Enzyme gel assays

*S*. *aureus* and *E*. *coli* DNA gyrase supercoiling and topo IV decatenation assay kits, as well as human topoisomerase II decatenation assay kits, were provided by Inspiralis (Norwich, UK). Assays were performed according to the manufacturer’s instructions. The compounds were serially diluted (1:3 dilutions starting from a 300 μM final concentration) and assayed in a reaction mixture to obtain concentration-response curves in two different replicate experiments. The final concentration of DMSO in the assays was 1% (v/v). The reactions were terminated by the addition of 30 μl chloroform/isoamyl alcohol (26:1) and 30 μl Stop Dye (40% sucrose, 100 mM Tris HCl pH 7.5, 1 mM EDTA, 0.5 μg/ml bromophenol blue), after which the samples were loaded onto a 1.0% TAE (0.04 mM Tris acetate, 0.002 mM EDTA) gel that was then run at 80 V for 2 hours. DNA bands were visualized by ethidium bromide staining the gel for 10 minutes, which was then destained for 10 minutes in water and analysed using gel documentation equipment (Syngene, Cambridge, UK). The DNA in each band was quantitated using Syngene Gene Tools software. Raw gel data (fluorescent band volumes) were converted by the software to percentages of the control (the fully supercoiled or decatenated DNA band). The data were then analysed using SigmaPlot Version 12.3 (2013). The global curve fit non-linear regression tool was used to calculate the IC_50_ values. The results are expressed as the average values obtained after calculating the IC_50_ from each replicate run. Etoposide was used as a positive control.

### Bacterial strains

The bacterial reference strains used in this study were purchased from ATCC (American Type Culture Collection) *via* LGC Standards (Middlesex, UK). *E*. *coli* 7623 TolC-, *Haemophilus influenzae* APT00129 AcrA- and *K*. *pneumoniae* 1161486a AcrAB- strains were provided by Aptuit (Verona, Italy). Clinical MDR isolates were provided by Aptuit, the IHMA Europa Sàrl repository (Epalinges, Switzerland) and the Statens Serum Institute (Copenhagen, Denmark).

### Antibacterial susceptibility testing

MICs were determined by the broth microdilution method (the agar dilution method was used only for *N*. *gonorrhoeae*) according to CLSI guidelines [27, 28]. Compounds **1**-**6**were obtained as DMSO-soluble powders and used for MIC determinations according to the method suggested by the CLSI for preparing dilutions of water-insoluble antibacterial agents [29]. The MIC values of antibiotics used in clinical practice were interpreted according to the susceptibility interpretive criteria reported in the appropriate CLSI Tables (M100-S25, CLSI, 2015) [29].

The results obtained using clinical isolates are reported as MIC_50_ and MIC_90_ values.

### Determination of minimal bactericidal concentration

Minimal bactericidal concentrations (MBCs) were determined according to the procedure described by Knapp [30]. Viable counts were performed after plates were incubated at 37°C for 24 hours. The MBC was defined as the lowest concentration that reduced the viability of the initial bacterial inoculum by ≥99.9%.

### Time-kill assays

Time-kill experiments were performed according to the procedure reported by Knapp [30]. Three doubling concentrations of each molecule (1×, 2× and 4× MIC) were tested, with compound **1** additionally assayed at 8× and 16× MIC against *S*. *aureus* 29213. A drug-free control was included in each experiment. The bacterial cultures were standardized to obtain an initial inoculum that ranged from 5×10^5^ to 5×10^6^ CFU/ml. The cultures were incubated at 37°C in a shaking bath. At 0, 2, 4, 8 and 24-hour intervals, viable counts were performed by spotting (in triplicate) 10 μl aliquots of suitable dilutions of the cultures onto Mueller-Hinton agar (MHA, Oxoid, Basingstoke, UK) plates. After incubation at 37°C for 24 hours, the bactericidal activity, namely, the ability to reduce the initial inoculum by 3 log_10_ CFU/ml, was assessed for each tested concentration over time.

### Post-antibiotic effect (PAE)

The PAEs of ciprofloxacin and compounds **1** and **2** were calculated as reported by Huband [31] with some modifications. Briefly, logarithmically growing cultures of *E*. *coli* ATCC 25922, *S*. *aureus* ATCC 29213, and *S*. *aureus* ATCC BAA-1720, at a starting concentration of 1×10^6^ CFU/ml, were exposed to various concentrations of drugs (*i*.*e*., 1×, 4× and 16× the MIC) for 1 hour at 37°C. A drug-free control was included in each experiment. After being exposed to the drug for 1 hour, viable counts were conducted in triplicate by spotting aliquots (10 μl) of suitable dilutions onto MHA plates. The drugs were removed by diluting the bacterial cultures (1:1000) in Cation-Adjusted Mueller-Hinton Broth (CAMHB; Sigma-Aldrich). The diluted cultures were incubated in a shaking bath at 37°C. To monitor the ongoing suppression of bacterial growth over time (5 hours), aliquots from each broth culture were periodically collected, diluted and plated to perform subsequent viable counts (in triplicate). PAE was defined as the difference between the times required for cultures exposed and unexposed to drugs to increase by 1 log_10_ CFU/ml above the number of CFUs counted immediately after dilution (1:1000).

### Selection of resistant *S*. *aureus* mutants

*In vitro* selection of *S*. *aureus* ATCC 29213 and of *S*. *aureus* ATCC BAA-1720 mutants was performed using a single-step mutation method as reported by Drago [32] using compounds **1** and **2** at concentrations of 2, 4, 8, 16, and 32 times the MIC values of these compounds. The frequency of mutation was calculated in the *S*. *aureus* ATCC 29213 as the ratio between the number of colonies (CFU/ml) grown on antibiotic-containing plates and the number (CFU/ml) of colonies in the initial inoculum. The increments in the MICs of mutants with respect to the MIC value of the parental strain were determined after 5 passages on MHA containing the same concentration of antibiotic used for selection and after 5 subsequent passages on drug-free MHA.

### Characterization of acquired resistance

*S*. *aureus* BAA-1720 drug-resistant mutants were analysed for the presence of point mutations in *gyrA* and *gyrB* (encoding the GyrA and GyrB gyrase subunits) and *grlA* and *grlB* (encoding the ParC and ParE topo IV subunits). Bacterial DNA from the parental and mutant strains was obtained according to the method described by Hynes [33]. PCR was performed in a final reaction volume of 50 μl, and the amplification programme reported by Huband [31] was used. The primer pairs (gyrA-F1 and gyrA-R1; gyrB-F1 and gyrB-R1; parC_grlA-F1 and parC_grlA-R1; and parE_grlB-F1 and parE_grlB-R1.1) used in PCR assays to amplify the genes encoding DNA gyrase (*gyrA* and *gyrB*) and topoisomerase IV (*grlA* and *grlB*) were previously reported by Huband [31]; the sequences (5’-3’) of the gyrA2_F and grlA-parC2_F primers are AGTGAATAAGGCTCGTATGA and CGTGATGAAACTGATAGAACT. The purified PCR products were sequenced (GATC Biotech Cologne, Germany), and the *gyrA*, *gyrB*, *grlA* and *grlB* gene sequences of the mutant strains were compared with those of the parental strain to detect the presence of point mutations. Nucleotide sequence analyses and comparisons were conducted using the Basic Local Alignment Search Tool (BLAST; http://blast.ncbi.nlm.nih.gov/Blast.cgi).

### *In vitro* preliminary ADME and tolerability assays

*Cytotoxicity assay*. Cytotoxicity of compounds **1** and **2** was assessed [34], after 24 hours of incubation in EMEM containing 10% fetal bovine serum, HepG2 mammalian cell line viability was evaluated using CellTiter-Blue Cell Viability Assay (Promega, WI, USA) according to the manufacturer’s instructions. Concentration-response curves were obtained by non-linear regression analysis using the GraphPad PRISM 6 software.

*Inhibition of P450 isoenzymes*. The inhibition of the activities of cytochrome P450 isoenzymes (CYP1A, CYP2B6, CYP2C8, CYP2C9, CYP2C19, CYP2D6 and CYP3A4) by compounds **1** and **2** was measured according to a standardized protocol [35].

*hERG automated patch clamp assay*. The human recombinant ERG CHO-K1 cell line was used in an automated patch clamp assay (qPatch system) to evaluate the hERG channel blockade potential of compounds **1** and **2** [36]. Concentration (log) response curves were fitted to a logistic equation to generate estimates of the 50% inhibitory value.

## Results

### Inhibition of target activity *in vitro*

Compounds **1**–**6** exhibited strong simultaneous inhibition of DNA gyrase and topo IV from *E*. *coli* and *S*. *aureus* (Table 1). Ciprofloxacin was selected as a representative quinolone and tested in parallel for comparison. The degree of inhibition against the both *E*. *coli* enzymes by these compounds was essentially equivalent, and greater than that observed with ciprofloxacin. The potent *in vitro* inhibition of both DNA gyrase and topo IV from *E*. *coli* and *S*. *aureus* was consistent with the hypothesized dual-targeting structural features of the six compounds. In comparison, the calculated ratio between the IC_50_ for human topoisomerase II and their bacterial counterparts (both DNA gyrase and topo IV) showed that this series of compounds is selective for bacterial topoisomerases (>32-fold difference) (Table 1; S2 File). The IC_50_ value for etoposide, the human topoisomerase II inhibitor used as a positive control, was 154 μM, consistent with literature data [37, 38].

**Table 1:** Dual inhibition of *E*. *coli* and *S*. *aureus* DNA gyrase and topoisomerase IV enzymes.

	IC_50_ (μM)				
Compound	*E*. *coli* Gyrase Supercoiling	*E*. *coli* Topoisomerase IV decatenation	*S*. *aureus* Gyrase Supercoiling	*S*. *aureus* Topoisomerase IV decatenation	Human Topoisomerase II decatenation
**1**	0.25	1.38	0.28	2.25	98
**2**	0.05	0.13	<0.1	<0.1	6
**3**	<0.1	<0.1	<0.1	<0.1	56
**4**	0.13	0.30	1.00	0.22	>300
**5**	0.10	0.14	0.17	0.07	9
**6**	0.13	0.25	0.31	0.29	10
ciprofloxacin	0.87	11.47	22.62	6.62	>300

### Bacterial susceptibility profiles

The antibacterial activity of the six selected compounds was first determined against *S*. *aureus* ATCC 29213 and *E*. *coli* ATCC 25922 to confirm the inhibition observed in the functional enzymatic assays. As shown in Table 2, all the compounds demonstrated strong effects against *S*. *aureus*, with MIC values equal to or lower than 0.5 μg/ml. In particular, compounds **2** and **6** showed MIC values that were approximately 10-fold lower than that of ciprofloxacin (MIC = 0.5 μg/ml). When tested against *E*. *coli* ATCC 25922, compounds **1**–**5** showed MIC values of 0.5 μg/ml, and compound **6** had an MIC of 0.25 μg/ml.

**Table 2:** Susceptibility of reference bacterial strains and mutant strains with impaired efflux pumps to ciprofloxacin and NBTI compounds.

	MIC (μg/ml)						
Strain	Ciprofloxacin	1	2	3	4	5	6
Ciprofloxacin-susceptible							
*E*. *coli* ATCC 25922	0.008	0.5	0.5	0.5	0.5	0.5	0.25
*S*. *aureus* ATCC 29213	0.5	0.25	0.03	0.25	0.25	0.5	≤0.06
*S*. *pneumoniae* ATCC 49619	1	0.5	≤0.06	0.25	0.25	0.5	0.12
*S*. *pyogenes* APV00083	0.5	1	≤0.06	0.12	0.12	≤0.06	-
*S*. *pyogenes* APV00074	0.25	0.5	≤0.06	≤0.06	0.12	0.12	-
*K*. *pneumoniae* ATCC 700603	0.25	4	>8	2	4	8	8
*H*. *influenzae* ATCC 49766	≤0.06	0.25	0.5	0.12	0.12	0.12	0.5
*P*. *aeruginosa* ATCC 27853	0.25	2	2	4	2	4	4
*N*. *gonorrhoeae* ATCC 19424	≤0.06	≤0.06	≤0.06	≤0.06	≤0.06	≤0.06	0.03
*N*. *gonorrhoeae* ATCC 49226	0.004	0.06	0.06	0.016	0.06	0.125	0.03
*A*. *baumannii* ATCC 19606	0.5	≤0.06	0.12	≤0.06	0.5	1	-
*E*. *faecalis* ATCC 29212	1	2	0.25	0.12	0.5	0.25	0.25
Ciprofloxacin-resistant							
*E*. *coli* 4SI	>8	0.25	0.5	0.12	0.25	0.5	-
*S*. *aureus* BAA-1720	128	0.5	0.016	0.12	0.5	1	≤0.06
*N*. *gonorrhoeae* WHO K	>8	0.12	0.12	0.016	0.12	0.25	0.06
*N*. *gonorrhoeae* WHO L	>8	0.12	0.06	0.016	0.12	0.25	0.03
*N*. *gonorrhoeae* WHO N	8	0.06	0.03	0.016	0.06	0.12	0.03
*A*. *baumannii* APV00473	>8	≤0.06	0.5	≤0.06	0.12	0.5	-
others							
*C*. *difficile* ATCC 700057	ND*	1	0.25	0.25	-	0.25	0.5
With impaired efflux pump							
*E*. *coli* TolC- mutant	≤0.016	≤0.016	≤0.016	≤0.016	≤0.016	≤0.016	≤0.016
*H*. *influenzae* AcrA- mutant	≤0.016	≤0.016	≤0.016	0.06	≤0.016	≤0.016	-
*K*. *pneumoniae* AcrAB- mutant	≤0.016	0.03	≤0.016	≤0.016	0.03	0.03	-

*Metronidazole MIC: 0.25 μg/ml

The spectrum of antibacterial activity of these compounds was assessed against a broad collection of reference bacterial strains, including several drug-resistant microorganisms (Table 2). Notably, all the compounds yielded lower MIC values than ciprofloxacin when tested against fluoroquinolone-resistant strains with MIC values comparable to those obtained against the ciprofloxacin-susceptible *E*. *coli* ATCC 25922 and *S*. *aureus* ATCC 29213 strains.

The tested compounds also exerted antibacterial effects against other clinically relevant Gram-positive (*S*. *pneumoniae* ATCC 49619, *E*. *faecalis* ATCC 29212, and *S*. *pyogenes* APV00083 and APV00074) and Gram-negative bacteria. In particular, both fluoroquinolone-susceptible (ATCC 19424 and ATCC 49226) and fluoroquinolone-resistant (WHO K, L and N) strains of *N*. *gonorrhoeae*, as well as ciprofloxacin-sensitive (ATCC 19606) and ciprofloxacin-resistant (APV00473) strains of *A*. *baumannii* were susceptible to our compounds.

The six NBTIs showed lower activity than ciprofloxacin against *H*. *influenzae* ATCC 49766, *K*. *pneumoniae* ATCC 700603 and *P*. *aeruginosa* ATCC 27853 (MICs ranging from 2 to >8 μg/ml). High susceptibility of the *E*. *coli* TolC^-^, *H*. *influenzae* AcrA^-^ and *K*. *pneumoniae* AcrAB^-^ mutant strains, which are deleted for one of the efflux pump protein complexes [39–41], suggests that the effectiveness of the test compounds is limited by efflux systems in these bacteria, since lower MIC values than those determined for non-mutant strains were observed (Table 2).

To verify the clinical relevance of the above results, the activity of the compounds against multidrug-resistant (MDR) clinical isolates was assessed (Table 3).

**Table 3:** Susceptibility data for clinical isolates of multidrug-resistant strains.

Organism (no. isolates)	Antibiotic	MIC (μg/ml)		
		range	MIC_50_	MIC_90_
*S*. *aureus* (n = 10)	**1**	0.12–0.5	0.12	0.5
	**2**	≤0.06	≤0.06	≤0.06
	**3**	≤0.06	≤0.06	≤0.06
	**4**	≤0.06–0.5	0.12	0.5
	**5**	≤0.06–0.5	0.12	0.5
	**6**	-	-	-
	ciprofloxacin	8->8	8	8
	vancomycin	1–4	1	2
*C*. *difficile* (n = 21)	**1**	0.25–2	1	1
	**2**	0.06–0.25	0.12	0.25
	**3**	0.06–0.25	0.12	0.25
	**4**	-	-	-
	**5**	0.06–1	0.5	1
	**6**	0.06–0.25	0.25	0.25
	metronidazole	0.25–2	0.5	1
	vancomycin	1–2	2	2
	clindamycin	4–32	8	32
	imipenem	2–16	8	16
	fidaxomicin	0.03–1	0.5	0.25
*E*. *faecium* (n = 12)	**1**	0.03–16	1	2
vanA (6)/vanB (6)	**2**	0.06–1	0.12	0.25
	**3**	0.06–2	0.12	0.25
	**4**	0.25–8	1	1
	**5**	0.25–8	1	4
	**6**	0.06–1	0.25	0.25
	ampicillin	>64	>64	>64
	penicillin	>64	>64	>64
	daptomycin	2–4	4	4
	vancomycin	>64	>64	>64
	teicoplanin	0.5->64	1	64
	linezolid	2	2	2
*E*. *faecalis* (n = 12)	**1**	0.03–0.25	0.03	0.25
vanA (6)/vanB (6)	**2**	0.06–0.12	0.12	0.12
	**3**	0.06–0.12	0.12	0.12
	**4**	0.12–1	0.25	0.5
	**5**	0.25–4	0.5	2
	**6**	0.06–0.12	0.12	0.12
	ampicillin	1–4	2	2
	penicillin	2–16	4	8
	daptomycin	0.5–2	1	1
	vancomycin	16->64	>64	>64
	teicoplanin	0.5>64	>64	>64
	linezolid	1–2	2	2
*E*. *coli* (n = 36)	**1**	0.25–16	1	1
	**2**	0.5–4	1	2
	**3**	0.25–4	1	2
	**4**	-	-	-
	**5**	0.5–16	2	4
	**6**	0.25–4	2	4
	ceftriaxone	0.03–32	1	32
	levofloxacin	0.03–32	4	16
	ampicillin	2->64	>64	>64
	colistin	0.5->16	0.5	0.5
	gentamicin	0.5->32	2	32
	tetracycline	1->64	16	>64
	imipenem	0.06–16	0.12	4
*A*. *baumannii* (n = 10)	**1**	≤0.06–0.12	0.12	0.12
	**2**	≤0.06–0.12	≤0.06	0.12
	**3**	≤0.06–0.12	≤0.06	0.12
	**4**	≤0.06–0.5	0.25	0.5
	**5**	0.25–1	0.5	1
	**6**	-	-	-
	ciprofloxacin	012->8	>8	>8
	meropenem	0.25->8	8	8
*N*. *gonorrhoeae* (n = 38)	**1**	0.016–0.12	0.12	0.12
	**2**	0.004–0.12	0.06	0.12
	**3**	0.004–0.03	0.016	0.03
	**4**	0.004–0.12	0.06	0.12
	**5**	0.016–0.25	0.12	0.25
	**6**	0.008–0.06	0.03	0.06
	ciprofloxacin	0.004->8	8	>8
	ceftriaxone	≤0.002–0.12	0.012	0.094

Among the Gram-positive pathogens, the molecules showed MIC_90_ values against ciprofloxacin-resistant *S*. *aureus* that ranged from ≤0.06 to 0.5 μg/ml, while the MIC_90_ of ciprofloxacin was measured as 8 μg/ml. Furthermore, the test compounds showed a higher potency than vancomycin.

Antibacterial activity was also observed against *C*. *difficile* strains, with an MIC_90_ that ranged from 0.25 to 1 μg/ml. The molecules showed an antibacterial effect against *C*. *difficile* stronger than vancomycin (MIC_90_ = 2 μg/ml) and comparable to those of metronidazole (MIC_90_ = 1 μg/ml) and fidaxomicin (MIC_90_ = 0.25 μg/ml).

Against vanA/vanB-mutant *E*. *faecium* isolates, observed MIC_90_ and MIC_50_ values for our hit compounds were much lower than those observed for the comparator antibiotics ampicillin, penicillin, vancomycin and teicoplanin, as well as daptomycin and linezolid. Similar effects were observed with the *E*. *faecalis* resistant isolates, against which the comparator antibiotics ampicillin, penicillin, daptomycin, vancomycin, teicoplanin and linezolid showed poor activity.

Against Gram-negative pathogens, the test compounds exhibited MIC_90_ values against *E*. *coli* MDR isolates, that were comparable to or even lower than those of other antibiotics, such as ceftriaxone, levofloxacin, ampicillin, colistin, gentamicin, tetracycline and imipenem.

Notably, the compounds displayed activity against *A*. *baumannii*, with observed MIC_90_ values ranging from 0.12 to 1 μg/ml, a very promising result compared to those for meropenem and ciprofloxacin (MIC_90_ ≥8 μg/ml).

Finally, the compounds were also tested on a panel of clinical *N*. *gonorrhoeae* isolates, with observed MIC_90_ and MIC_50_ values comparable to those obtained with the clinical standard ceftriaxone, while ciprofloxacin showed a poor activity (Table 3).

### Determination of minimal bactericidal concentrations

Compounds **1** and **2** were further studied to better characterize their antibacterial activity and drug-like properties. Compound **1** was selected based on its unique chemical structure compared to the other five compounds (discontinuity of the aromaticity in the bicyclic region). Compound **2** was chosen as the best representative of the class based on its overall antibacterial profile. The MBCs of these compounds were evaluated against the strains *E*. *coli* ATCC 25922, *S*. *aureus* ATCC 29213 and *S*. *aureus* ATCC BAA-1720 (Table 4). Although compounds **1** and **2** produced lower MIC values against both *S*. *aureus* strains than against *E*. *coli* ATCC 25922, compound **2** showed lower bactericidal activity against *S*. *aureus* (MBC/MIC ratios of ≥16) compared to that observed against *E*. *coli* (MBC/MIC ratios of 2). The test confirmed the very low susceptibility of the *S*. *aureus* ATCC BAA-1720 strain to ciprofloxacin.

**Table 4:** Minimal bactericidal concentration (MBC) values of selected compounds against *E*. *coli* ATCC 25922, *S*. *aureus* ATCC 29213 and *S*. *aureus* BAA-1720.

Compound	Strain	MIC(μg/ml)	MBC(μg/ml)	MBC/MIC
Ciprofloxacin	*E*. *coli* ATCC 25922	0.008	0.016	2
	*S*. *aureus* ATCC 29213	0.5	1	2
	*S*. *aureus* BAA-1720	128	256	2
Compound **1**	*E*. *coli* ATCC 25922	0.5	1	2
	*S*. *aureus* ATCC 29213	0.25	4	16
	*S*. *aureus* BAA-1720	0.5	1	2
Compound **2**	*E*. *coli* ATCC 25922	0.5	1	2
	*S*. *aureus* ATCC 29213	0.03	0.5	16
	*S*. *aureus* BAA-1720	0.016	2	125

### Time-kill studies

**Fig 2:**
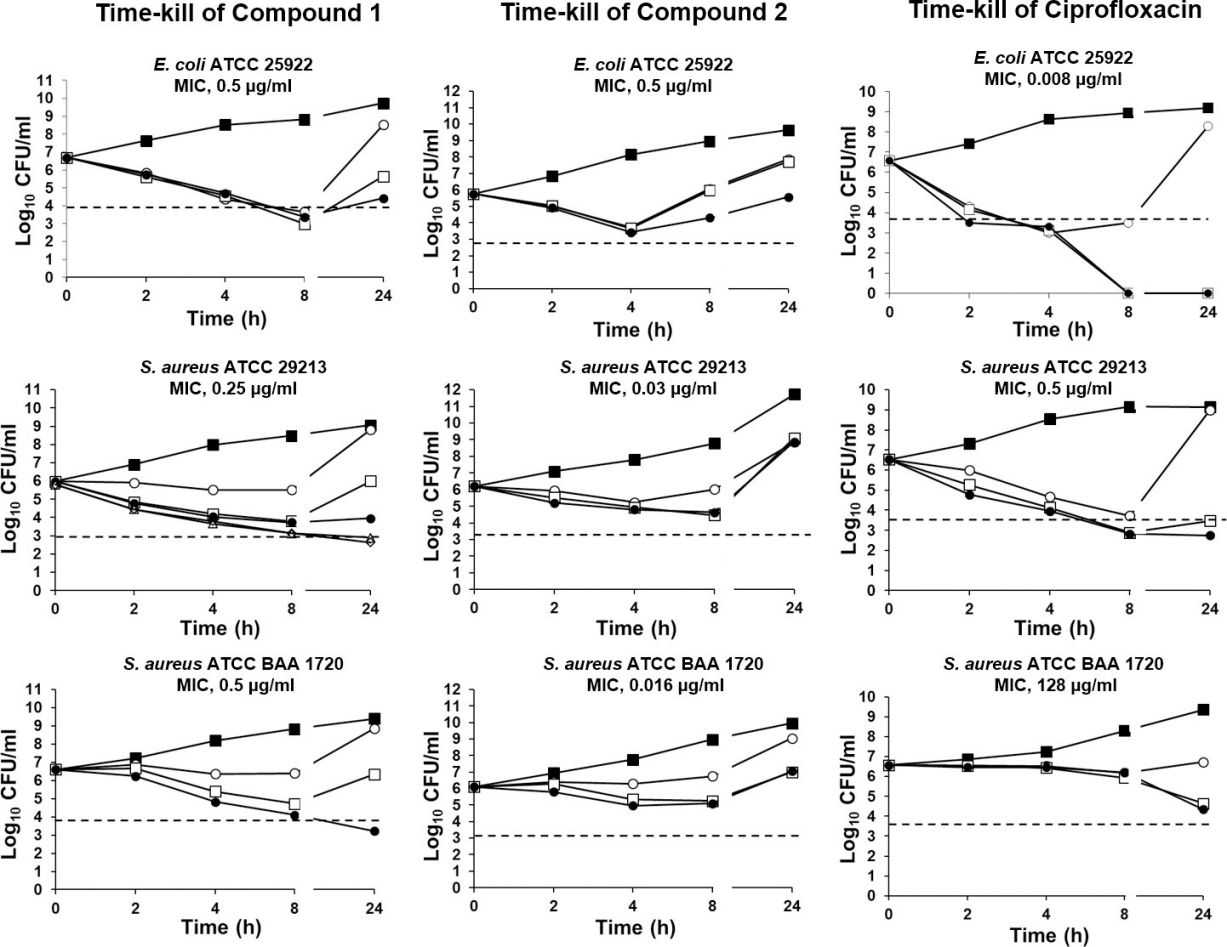
Bactericidal activities of compounds 1 and 2 and ciprofloxacin against *E*. *coli* ATCC 25922, *S*. *aureus* ATCC 29213, and *S*. *aureus* ATCC BAA-1720. All experiments were performed in triplicate. The threshold corresponding to a reduction of 3 log_10_ CFU/ml is indicated by the black dashed line. The standard deviations of all the reported values are less than 0.5 log_10_. Bacteria were grown in the presence of the indicated compound concentrations: ■ no drug; ○ 1× MIC; □ 2× MIC; ●, 4× MIC; Δ 8× MIC; ◊ 16× MIC.

Fig 2 shows the *in vitro* time-kill activity of ciprofloxacin and of compounds **1** and **2** against *E*. *coli* ATCC 25922, *S*. *aureus* ATCC 29213 and *S*. *aureus* ATCC BAA-1720. Ciprofloxacin exhibited no bactericidal effect against *S*. *aureus* ATCC BAA-1720. Notably, compound **1** showed a bactericidal effect against all three strains. A reduction in viable cell count was observed for *E*. *coli* ATCC 25922 at all tested concentrations. Additionally, bactericidal activity against the methicillin-resistant strain *S*. *aureus* ATCC BAA-1720 was observed at 2 μg/ml (4× MIC) after a 24 hour incubation. Since concentrations up to 4× MIC resulted in no bactericidal activity against *S*. *aureus* ATCC 29213, two additional concentrations (8× and 16× MIC) were tested, which were effective after 24 hour incubation. In contrast, compound **2** displayed no bactericidal activity against any of the strains at any of the tested concentrations (from 1× to 4× MIC) over the 24-hour incubation period. Both compounds showed slower killing kinetics than those observed for ciprofloxacin, and regrowth after 24 hours was observed in the presence of antibiotic concentrations lower than or equal to the MBC in all three bacterial strains. Regrowth after 24 hours is not an uncommon observation, and it has also been reported for ciprofloxacin and other NBTIs [23, 42, 43].

### PAE testing

**Fig 3:**
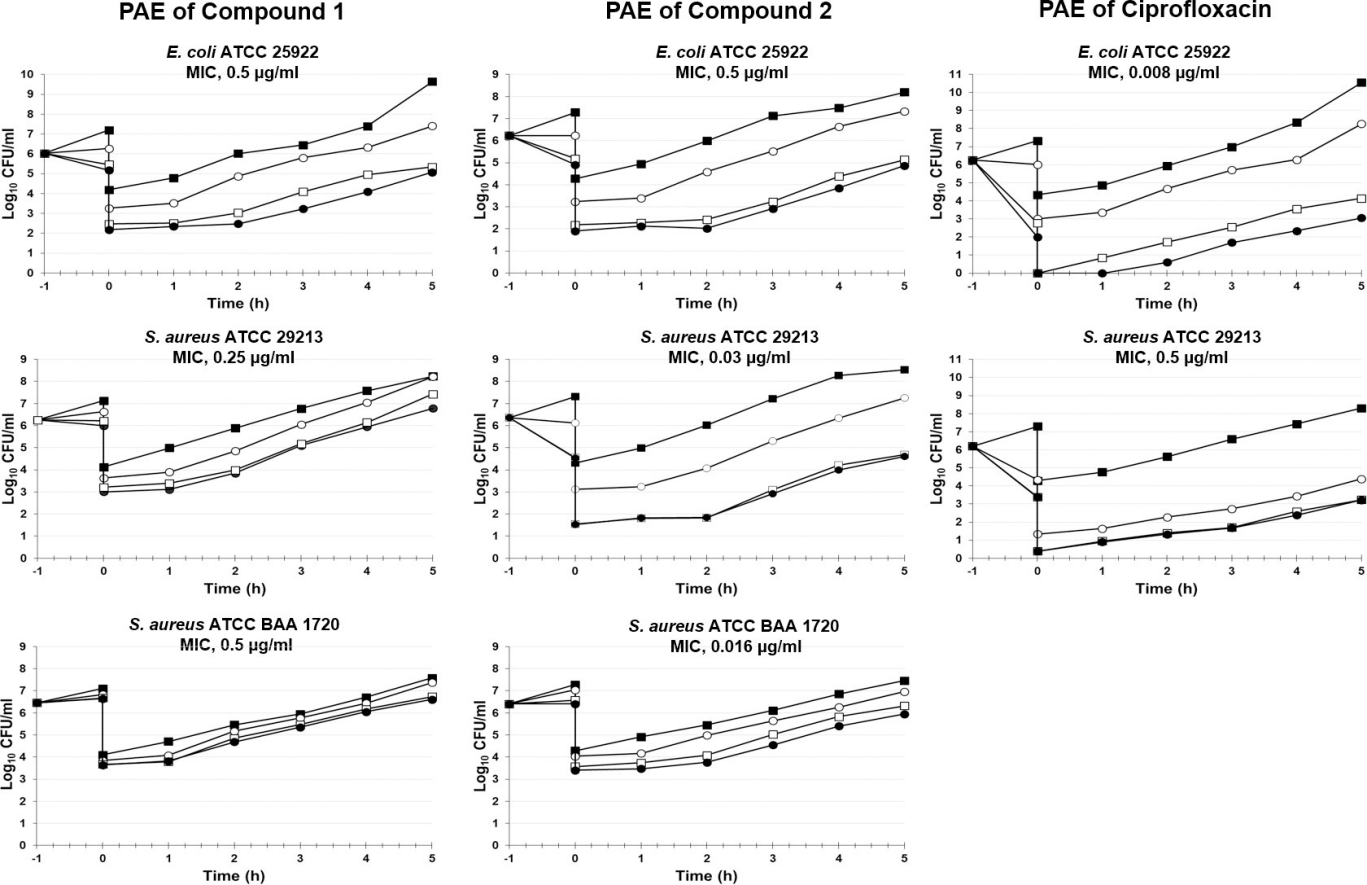
Post-antibiotic effect (PAE) induced by a 1-hour exposure of *E*. *coli* ATCC 25922, *S*. *aureus* ATCC 29213 and *S*. *aureus* ATCC BAA-1720 to compounds 1 and 2 and ciprofloxacin. The PAE induced by ciprofloxacin was not assessed in the *S*. *aureus* BAA-1720 strain due to its resistance to ciprofloxacin (MIC = 128 μg/ml). All experiments were performed in triplicate. The standard deviations of all the reported values are less than 0.5 log_10_. The bacteria were grown in the presence of the indicated compound concentration: ■ no drug; ○ 1× MIC; □ 4× MIC; ● 16× MIC.

The term post-antibiotic effect (PAE) is used to describe the suppression of bacterial growth that persists after a short exposure of organisms to antibacterials [44]. The PAE testing results are shown in Fig 3. Persistent suppression of bacterial growth after antibacterial exposure appeared to be a feature of both tested compounds against all organisms. The duration of PAEs differed for specific compound/microorganism combinations, and increasing concentrations were associated with a progressive increase in PAEs. The PAEs of compounds **1** and **2** against *E*. *coli* ATCC 25922 at 1× and 4× MIC were consistently higher than those observed for ciprofloxacin, and in particular, compound **2** showed PAE values that were approximately two-fold greater than those obtained for ciprofloxacin. Ciprofloxacin also showed short PAEs against *S*. *aureus* ATCC 29213, with PAE values ranging from 25 minutes (at 1× and 4× MIC) to 35 minutes (16× MIC), whereas the relevant PAEs of compounds **1** and **2** were two-fold (54–58 min) and three-fold (73–78 min) greater than those observed for ciprofloxacin, respectively.

The PAE values of the two NBTIs against *S*. *aureus* ATCC BAA-1720 were shorter than those observed for *S*. *aureus* ATCC 29213, with compound **2** being more effective than compound **1** in the suppression of bacterial growth after its removal from broth culture. The PAE values of compound **1** ranged from 10 to 25 minutes, whereas those of compound **2** ranged from 20 to 70 minutes.

### Spontaneous resistance frequencies

Table 5 shows the *in vitro* frequencies of resistant mutants after the exposure of *S*. *aureus* ATCC 29213 to compounds **1** and **2** and to ciprofloxacin. The resistant mutants were obtained via a single-step mutation method at antibacterial concentrations ranging from 2× to 32× MIC. For compound **1**, the mutation frequency of *S*. *aureus* ATCC 29213 was 1.4×10^−7^ at 4× MIC (1 μg/ml), while for compound **2**, the mutation frequency was 6.7×10^−7^ at 2× MIC (0.06 μg/ml). At higher concentrations, neither compound resulted in the selection of resistant mutants (mutation frequency lower than 1×10^−8^). In comparison, ciprofloxacin-resistant mutants of *S*. *aureus* ATCC 29213 were obtained on agar containing 1 μg/ml (2× MIC) ciprofloxacin at a mutation frequency of 4×10^−8^. Mutants resistant to both compounds showed increases in MICs of up to 32-fold relative to the MIC values of the parental strains.

**Table 5:** *In vitro* frequency of mutants resistant to ciprofloxacin and to compounds 1 and 2 in *S*. *aureus* ATCC 29213.

	*S*. *aureus* ATCC 29213		
MIC-fold	ciprofloxacin	1	2
**2×MIC**	4 ∙ 10^−8^	TNTC	6.7 ∙ 10^−7^
**4×MIC**	<1 ∙ 10^−8^	1.4 ∙ 10^−7^	<1 ∙ 10^−8^
**8×MIC**	<1 ∙ 10^−8^	<1 ∙ 10^−8^	<1 ∙ 10^−8^
** 16×MIC**	<1 ∙ 10^−8^	<1 ∙ 10^−8^	<1 ∙ 10^−8^
**32×MIC**	<1 ∙ 10^−8^	<1 ∙ 10^−8^	<1 ∙ 10^−8^

### Analysis of *S*. *aureus* BAA-1720 mutants

To investigate the occurrence and distribution of point mutations, the *S*. *aureus* ATCC BAA-1720 mutant strains displaying the highest increases in MIC values for both compounds (16- or 32-fold MIC) were selected for sequencing analysis of the *gyrA*, *gyrB*, *grlA*, and *grlB* genes. Sequencing analysis of the *grlA* gene in the parental strain revealed a change resulting in the substitution of serine for phenylalanine at amino acid position 80, corresponding to a well-known point mutation within the quinolone resistance-determining region (QRDR) that confers fluoroquinolone resistance. This substitution was also detected in the *S*. *aureus* ATCC BAA-1720 mutant isolates.

The point mutations observed after exposure of the bacteria to compounds **1** and **2** occurred in the two genes encoding the DNA gyrase. The mutant resistant to compound **1** showed a point mutation in *gyrB* resulting in the replacement of a proline at amino acid position 456 by leucine. The substitution observed in the mutant resistant to compound **2** occurred at position 44 in gyrA subunit, outside of the QRDR, causing a proline to be replaced with a leucine residue. Interestingly, these two mutations have been already described as induced by other NBTI compounds [45] confirming the NBTI nature of our molecules.

The resistance to compounds **1** and **2** in *S*. *aureus* ATCC BAA-1720 was associated with a fitness cost as demonstrated by the reduced bacterial growth rate of the mutants (55% and 30% reduction in mutants resistant to compound **1** and to compound **2**, respectively) S3 File.

### *In vitro* safety profile

The two selected compounds were preliminarily characterized using a panel of *in vitro* ADME-Tox assays. The IC_50_ values for the effects of compounds **1** and **2** on mammalian cell viability were observed to be 9 μM (4 μg/ml) for compound **1** and >300 μM (>145 μg/ml) for compound **2**; these values are higher than the corresponding MIC values observed against the tested pathogens. However, this result was quite surprising because compound **2** is less specific for the bacterial vs. human topoisomerase enzyme than compound **1**, that consequently was expected to exert a lower cytotoxicity. It could be hypothesized that compound **1** has a higher permeability through mammalian cell membrane and therefore reaches higher intracellular concentrations; alternatively, compound **1** could interact with off-target intracellular pathways involved in cellular viability.

Compounds **1** and **2** were devoid of inhibitory activity against all the CYP450 isoenzymes tested (% inhibition ≤35% at 10 μM) with the exception of CYP2C19, against which they showed 52 and 69% inhibition, respectively. Moreover, in an automated patch clamp assay, the measured IC_50_ against hERG channels was 8.9 μM for compound **1** and >100 μM for compound **2**.

Since the compounds displayed high affinity for plasma proteins (>99.9% protein binding), MIC values against *S*. *aureus* ATCC 29213 and *S*. *aureus* BAA-1720 were obtained in the presence of 20% human serum to elucidate the relevance of this feature to compound evaluation. The antibacterial activity of compound **2** was not modified by the addition of serum whereas compound **1** showed a two-fold increase of the MIC value.

## Discussion

The increasing rate of bacterial resistance to antibiotics and its impact on the treatment of infectious diseases have raised public health concerns of growing importance [2]. Together with measures that decrease the rate of emergence of novel resistance mechanisms by limiting the misuse of antibacterials, new and more effective antibiotics with innovative mechanisms of action are urgently needed in medical practice [5]. Recently, it was reported that the activity of bacterial DNA gyrase and topo IV, clinically validated targets of the quinolone class of antibiotics, [10, 11] can be blocked by NBTI molecules that target a site that is distinct from the quinolone-binding sites [19]. Drugs that possess such a mode of action could be used to overcome bacterial resistance to quinolones.

To this end, we initiated a discovery programme that allowed the identification of a series of novel compounds belonging to the NBTI class [25]. These compounds may therefore represent a new chemical series of clinically relevant antibacterial agents that act on bacterial DNA gyrase and topo IV.

The newly discovered N-linked piperazine core was a suitable starting point for further development of new and more potent NBTIs [25]. The optimization strategy focused on modifying the RHS, LHS groups and linker of the initial series was carried out, providing compounds **1**–**6**. Particularly, the new series was obtained combining: i) the RHS 8-fluoro[1]benzopyrano[4,3-b]pyrrol-4(1H)-one which was derived by cyclization of the 4-aminocoumarin ring previously explored [25]; ii) different saturated and partially unsaturated LHS oxazinones; iii) and N-ethyl-4-methylcyclohexan-1-amine linker.

The resulting compounds showed a balanced sub micromolar activity against the two enzymes isolated from *S*. *aureus* and *E*. *coli* strains with the exception of compound **1**. Specifically, compound **1** resulted from 5 to 22-fold less potent against topo IV in both strains. The lower activity could be due to the different LHS contribution to the DNA binding, depending on the aromatic content of the LHS ring. Indeed, the partially unsaturated heterocyclic LHS 7-fluoro-1H-pyrido[2,3-b][1,4]oxazin-2(3H)-one of compound **1** might form less tight π-π stacking interaction with the DNA bases than the aromatic LHS groups of compounds **2**–**6**, resulting in a substantial loss of the enzymatic inhibition. Conversely, the conserved RHS ring contributed to the activity penetrating into the hydrophobic cavity created by the two GyrA subunits and occupying the deepest part of the pocket helped by the stretched linker. The neighbouring residues Ala68, Val71, Met75 and Met121 contributed to the Van der Waals interactions with the 8-fluoro[1]benzopyrano[4,3-b]pyrrol-4(1H)-one group to further stabilize the ternary complex [25].

Consistent with their proposed mechanism of action, the six compounds exerted antibacterial activity against both Gram-positive and Gram-negative ciprofloxacin-resistant reference strains. It is noteworthy that the resistant strain *S*. *aureus* ATCC BAA-1720 was highly susceptible to the novel compounds despite its fluoroquinolone resistance. Moreover, in terms of bacterial growth inhibition, the compounds were effective against *S*. *aureus* strains and other Gram-positive bacteria, such as *S*. *pneumoniae*, *E*. *faecalis*, *C*. *difficile* and *S*. *pyogenes*.

In some Gram-negative organisms, such as *H*. *influenzae*, *K*. *pneumoniae* and *P*. *aeruginosa*, the series of compounds showed limited activity, with MIC values that were higher than those obtained for ciprofloxacin. The activity of all the molecules was greatly enhanced when tested against mutant strains lacking efflux pumps. The increased susceptibility of mutant strains to the novel molecules is therefore likely related to the modified efflux rate of bacteria. This is a key aspect mainly for those bacteria, such as *K*. *pneumoniae*, for which efflux pumps are encountered with a high frequency in the microbial population [46, 47].

Because of the potential of these molecules to serve as broad-spectrum antibacterial agents, they were assayed against a collection of clinically relevant bacterial isolates with multiple antibiotic resistances.

The susceptibilities of the *S*. *aureus* isolates to the six compounds was greater than those observed for ciprofloxacin and vancomycin. Moreover, activity of compounds **1** and **2** against a collection of clinical MRSA and macrolide resistant *S*. *aureus* isolates was also observed (S4 File). These data are of great interest, since MRSA is one of the leading causes of nosocomial infections [48, 49]. The results obtained against *E*. *faecium* and *E*. *faecalis* are also of particular significance, since our compounds showed MICs against these species that were definitively lower than those of the comparator antibiotics. This finding is important because enterococci, although normal components of the human intestinal flora, may occasionally become agents of infection, especially in hospitalized patients [50]. Compounds active against both species are attractive because they also inhibit the growth of VanA/VanB enterococci.

Infections caused by Gram-negative bacteria pose a great challenge due to difficulties in overcoming emerging resistance among these pathogens (multidrug resistance and pandrug resistance phenomena), thus precluding even the use of the most effective drugs [51]. Although discussed compounds display low activity against *H*. *influenzae*, *K*. *pneumoniae* and *P*. *aeruginosa*, they displayed a potent antibacterial activity against *E*. *coli*, *A*. *baumannii* and *N*. *gonorrhoeae*, providing a promising option for the treatment of such Gram-negative pathogens, which are often multidrug-resistant and difficult to eradicate [52, 53].

Compounds **1** and **2** were selected for further characterization of their antibacterial activities and to assess their *in vitro* safety profiles. The kinetics of killing revealed that compound **1** was bactericidal against all tested strains within 24 hours, exhibiting higher activity than ciprofloxacin. Surprisingly, compound **2**, for which the MIC values against the same strains were very low, was not bactericidal in time-kill experiments at any of the tested time points or concentrations. Regrowth after 24 hours was observed at low concentrations of the compounds, particularly in the case of compound **2** against *S*. *aureus* BAA-1720. This result could be related to the fact that the concentrations (1×, 2×, and 4× MIC, corresponding to 0.016, 0.03 and 0.06 μg/ml, respectively) used in the experiments had no bactericidal effect (MBC = 2 μg/ml).

PAE [39] after a 1 hour exposure to compounds **1** and **2**, against the same strains used in time-kill experiments, was greater than that observed for ciprofloxacin, and this effect was maintained against the quinolone-resistant *S*. *aureus* strain, particularly in the case of compound **2**. These findings, taken together with the high MBC/MIC ratio observed for compound **2** against *S*. *aureus* strains, suggested that this compound has bacteriostatic activity, particularly against staphylococci. However, the MIC and PAE results do not exclude compound **2** from being considered one of the most interesting compounds. Indeed, the clinical implication of increased PAEs includes the possibility of increasing the intervals between drug administration, thereby reducing treatment cost and decreasing selection for resistance [44, 54, 55].

The frequencies of the occurrence of spontaneous resistance in the *S*. *aureus* ATCC 29213 strain to compounds **1** and **2** were similar (1.4×10^−7^ and 6.7×10^−7^, respectively), although resistance developed at different antibacterial concentrations (4× and 2× MIC, respectively). At higher concentrations, the frequency of resistance was less than 1×10^−8^ for all compounds and comparable to ciprofloxacin. These data suggest a low potential for resistance development for the compounds in line with the balanced dual-targeting activity and are in agreement with recently published data describing new NBTIs [45, 56–58]. Tam *et al*. [56] generated *S*. *aureus* ATCC 29213 mutants that were resistant (mutation frequency of 3×10^−6^) to the compound AZD5206 at 3× MIC. Similarly, the NBTI compounds described by Lahiri *et al*. [45] and by Tan et al. [58] showed mutation frequencies of 1.7×10^−8^ and 6.7×10^−7^ at concentrations of 4× MIC. For Gram-negative strains, a mutation rate of 1.8×10^−7^ was reported in *P*. *aeruginosa* exposed to NBTI-5463 at 2× MIC [57]. Overall, the mutation rates observed with our compounds **1** and **2** are comparable to those observed for quinolones [31, 59, 60], although lower frequencies of resistance are reported for these compounds, particularly in Gram-negative bacterial strains [61].

The ciprofloxacin-resistant *S*. *aureus* ATCC BAA-1720 strain was used to differentiate the site of action of our novel NBTIs from that of quinolones. The finding that mutations induced by our compounds map outside the QRDR region and the susceptibility of the ciprofloxacin-resistant *S*. *aureus* ATCC BAA-1720 strain to our NBTI molecules strongly argues in favour of a site of action of our compounds distinct from the quinolones’ one. Moreover, the substitutions induced by our compounds are among those reported as induced also by other NBTIs [45] so that we can hypothesize that our compounds act in a similar way, that is by determining conformational changes in the DNA gyrase subunits affecting their function. The different bactericidal/bacteriostatic behaviour may be related to the different conformational changes induced by the two compounds.

Overall, considering the stage of the program, these data allow us to conclude that both compounds have a significant antibacterial efficacy with safety and physicochemical characteristics that can be further optimized. To this purpose, different LHS as well as RHS groups will be explored to modulate the lipophilicity of the molecule and accordingly improve ADMET properties, while potent antibacterial activity should be retained.

Overall, the present study demonstrates that our newly described NBTIs act on a clinically validated target through a mechanism of inhibition that confers to them the capacity to overcome resistance mechanisms and makes them active against quinolone-resistant bacterial strains. The significant antibacterial activity and the acceptable *in vitro* safety profile of these molecules, together with the potential lack of quinolone-like side effects due to their different chemical structure, justify further studies to optimize this series of compounds and makes them interesting leads in the development of new broad-spectrum antibacterial agents.

## Supporting information

S1 File Synthetic procedure of compounds 1 to 6. (DOCX)

S2 File Original figures of enzyme Gel assays. (PDF)

S3 File Growth rate of the wild type S. aureus ATCC BAA-1720 and of two resistant mutants. (PDF)

S4 File Susceptibility data of compounds 1 and 2 against a collection of S. aureus clinical isolates. (PDF)
