# Supplementary material for: Antibacterial activity of novel dual bacterial DNA type II topoisomerase inhibitors
Source: PLoS One. 2020 Feb 19;15(2):e0228509. doi: 10.1371/journal.pone.0228509 (PMC7029851; doi:10.1371/journal.pone.0228509)

**Synthetic procedure of compounds 1 to 6**

***General Information*.** Reagents and starting materials were purchased from commercial sources and used as received. Microwave-assisted reactions were carried out through a Biotage Initiator+ microwave synthesizer. Reaction progress was monitored by thin layer chromatography (TLC, Merck silica gel 60 F254 plates, 0.04−0.063 mm) or UPLC-QTOF chromatography (Waters SYNAPT MS-ACQUITY UPLC system).

Semi-preparative HPLC purification were run using a X-Bridge Prep C18 5 μm column (Waters) through Waters 2767 Sample manager, Waters 2478 dual λ absorbance detector, Waters Micromass ZQ single quadrupole mass spectrometer with an electrospray ionization (ESI) source. Samples were dissolved in DMSO/MeCN 1:1 (50 mg/mL). Purification was accomplished using a proper gradient of phase A (MeCN + 0.1% formic acid) and phase B (H_2_O + 0.1% formic acid) and a 40 mL/min flow rate at 25 °C. Fraction collection was managed by MassLynx software (v. 4.1).

Compound purity and molecular mass were determined by high-pressure liquid chromatography (HPLC) and high resolution mass spectrometry (UPLC-QTOF). HPLC analysis was carried out through a pump/autosampler (Waters Alliance 2695), a UV photo diode array detector (Waters 2996), and a Waters system data management (Empower 2), using Suplex pkb-100 (250 × 4.6 mm, 5 μm) column. For UPLC-QTOF, an Acquity BEH C18 (2.1 × 50 mm, 1.7 μm) column was used and a positive ion mode in the “VOptics” configuration was employed. Leucine-enkephalin (200 pg/μL) was used as the lock mass in order to provide authenticated exact mass measurement in MS and MS/MS modes within 5 ppm RMS mass accuracy. Nuclear Magnetic Resonance Spectroscopy (^1^H-NMR) were obtained using a Bruker Avance system (300, 400, and 500 MHz). All resonance bands were referenced to tetramethylsilane (TMS) as internal standard. For ^1^H-NMR spectroscopy: (s) = singlet; (d) = doublet; (t) = triplet; (q) = quartet; (quin) = quintet; (dd) = double doublet; (dt) = double triplet; (ddd) = double double doublet; (dtd) = double triple doublet; (m) = multiplet; (br) = broad; J = coupling constant (Hz); and δ = chemical shift (ppm).

***Synthesis of the key common intermediate 8-fluoro-4-oxo-1,4-dihydro[1] benzopyrano[4,3-b]pyrrole-2-carbaldehyde (Key Int.)***

The key intermediate *8-fluoro-4-oxo-1,4-dihydro[1]benzopyrano[4,3-b]pyrrole-2-carbaldehyde* (**Key Int.**) was prepared as described herein below, following the synthetic pathway A:

**Step 1**: POCl_3_ (25.8 mL) was added to DMF (40 mL) in one portion at 0°C. The resulting mixture was heated at 50°C for 0.5 h and a solution of 6-fluoro-4-hydroxycoumarin (10 g, 55.5 mmol) in DMF (30 mL) was added at 50°C. The reaction mixture was further heated at 60°C overnight, then concentrated in vacuo, treated with toluene (2 x 50 mL) and evaporated. DCM (400 mL) was added and the resulting mixture was poured onto ice and stirred for 10 min keeping the temperature around 0°C. The organic phase was separated, dried and evaporated in vacuo to obtain the crude product, 4‐chloro‐6‐fluoro‐2‐oxo‐2H‐chromene‐3‐carbaldehyde, which was progressed into the next step without further purification and characterization.

**Step 2**: 4‐chloro‐6‐fluoro‐2‐oxo‐2H‐chromene‐3‐carbaldehyde (12.5 g, crude material) and glycine ethyl ester hydrochloride (8.1 g, 58.3 mmol) were suspended in absolute EtOH (120 mL). TEA (3 eq.) was added at 0°C and the resulting mixture was stirred at the same temperature for 1 h and at 80°C for 24 h. The mixture was concentrated in vacuo, dissolved in DCM (400 mL) and washed with sat. NaHCO_3_ (200 mL). The organic phase was dried over Na_2_SO_4_ and evaporated in vacuo. The crude material was treated with EtOH (10 mL) to obtain 8-fluoro-4-oxo-1,4-dihydro[1]benzopyrano[4,3-b]pyrrole-2-carboxylate. Yield = 51%. LC-MS [M+H]^+^= 276.0.

**Step 3**: 8-fluoro-4-oxo-1,4-dihydro[1]benzopyrano[4,3-b]pyrrole-2-carboxylate (7.9 g, 28.7 mmol) was dissolved in dry THF (240 mL). The solution was chilled to -10°C then LiAlH_4_ (1 M solution in THF, 38.2 mL) was slowly added. The reaction mixture was stirred at 0°C for 4 h then was quenched by adding Na_2_SO_4_·10H_2_O. The inorganic salts were filtered off and the solvents were evaporated to recover 8-fluoro-2-(hydroxymethyl)[1]benzopyrano[4,3-b]pyrrol-4(1H)-one, that was progressed without any further purification. Yield = 48%. LC-MS [M+H]^+^ = 234.1.

**Step 4**: To a solution of 8-fluoro-2-(hydroxymethyl)[1]benzopyrano[4,3-b]pyrrol-4(1H)-one (1.2 g, 5.15 mmol) in DMSO (12 mL), Dess-Martin periodinane (2.4 g, 5.66 mmol) was added. The mixture was stirred at rt for 30 min then an aqueous solution of sat. NaHCO_3_/10% Na_2_S_2_O_3_ 1:1 was added. The precipitate was filtered, washed with water and treated with MeCN (4 mL) and diethyl ether (10 mL) to obtain the title key intermediate (**Key Int.**). Yield = 92%. LC-MS [M+H]^+^ = 232.1.

For the synthesis of final compounds, the further preparation of the 4-aminocyclohexyl group properly substituted in position 1, was set up following different strategies depending on the existing conjugated cyclic system. This portion was finally linked, using a common reaction, to the key intermediate 8-fluoro-4-oxo-1,4-dihydro[1]benzopyrano[4,3-b]pyrrole-2-carbaldehyde (**Key Int.**) to give each final compound.

***Synthesis of 8-fluoro-2-({[trans-4-(7-fluoro-2-oxo-2,3-dihydro-1H-pyrido[2,3-b][1,4]oxazin-1-yl)cyclohexyl]amino}methyl)[1]benzopyrano[4,3-b]pyrrol-4(1H)-one (Compound 1)***

The final Compound 1 was prepared as described herein below, following the synthetic pathway B:

**Step 1**: 5-Fluoro-3-nitropyridin-2-ol (10 g, 63.3 mmol) was dissolved in EtOH (300 mL), 10% Pd/C (1.8 g) was added and the mixture was stirred at room temperature under atmospheric pressure of hydrogen for 2 h. Pd/C was removed by filtration and the solvent was evaporated in vacuum to obtain the 3-amino-5-fluoropyridin-2(1H)-one as an off-white solid. Yield =92%. LC-MS [M+H]^+^ = 129.0.

**Step 2**: 3-amino-5-fluoropyridin-2(1H)-one (3.7 g, 28.9 mmol) and *tert*-butyl (4-oxocyclohexyl)carbamate (8 g, 37.6 mmol) were dissolved in DMF (213 mL). TFA (18.4 mL, 240 mmol) was added dropwise followed by NaBH(OAc)_3_ (9.19 g, 43.4 mmol). The mixture was stirred at room temperature for 1.5h then the reaction was quenched with sat. NaHCO_3_. The mixture was extracted with EtOAc, dried over Na_2_SO_4_ and evaporated under reduced pressure. The residue was purified by flash chromatography to give *tert*-butyl {4-[(5-fluoro-2-hydroxypyridin-3-yl)amino]cyclohexyl}carbamate as a formate salt. This material was dissolved in EtOAc, washed with sat. NaHCO_3_ sat. sol., dried over Na_2_SO_4_ and evaporated under reduced pressure to give the free base of the target compound as mixture of isomers. Yield = 60%. LC-MS [M+H]^+^ = 326.3.

**Step 3**: To a suspension of *tert*-butyl {4-[(5-fluoro-2-hydroxypyridin-3-yl)amino]cyclohexyl}carbamate (5.8 g, 17.8 mmol) and K_2_CO_3_ (9.9 g, 71.3 mmol) in DMF (110 mL) chloroacetyl chloride (3.1 mL, 39.2 mmol) was added. The mixture was stirred at room temperature for 30 min then heated to 80°C for 5 h and finally cooled to 0°C. Sat. NaHCO_3_ was added (50 mL) followed by EtOAc (200 mL). The organic phase was separated, dried over Na_2_SO_4_ and evaporated in vacuo. The crude product was purified by flash chromatography to obtain *tert*-butyl [4-(7-fluoro-2-oxo-2,3-dihydro-1H-pyrido[2,3-b][1,4]oxazin-1-yl)cyclohexyl]carbamate as a mixture of isomers. Yield = 58%. LC-MS [M+H]^+^ = 366.4.

**Step 4**: *tert*-butyl [4-(7-fluoro-2-oxo-2,3-dihydro-1H-pyrido[2,3-b][1,4]oxazin-1-yl) cyclohexyl]carbamate (3.79 g, 10.4 mmol) was dissolved in DCM (70 mL), TFA (10 mL) was added at 0°C then the mixture was stirred at rt for 2h. The solvent was evaporated in vacuo and the residue was first purified by chromatography and then by preparative HPLC, under basic conditions, to afford the diastereoisomer 1-(trans-4-aminocyclohexyl)-7-fluoro-1H-pyrido[2,3-b][1,4]oxazin-2(3H)-one. Yield = 41%. LC-MS [M+H]^+^ = 266.0.

**Step 5**: 8-fluoro-4-oxo-1,4-dihydro[1]benzopyrano[4,3-b]pyrrole-2-carbaldehyde (**Key Int**.: 158 mg, 0.41 mmol) and 1-(trans-4-aminocyclohexyl)-7-fluoro-1H-pyrido[2,3-b][1,4]oxazin-2(3H)-one (100 mg, 0.37 mmol) were suspended in dry DCM (20 mL). 2 drops of acetic acid were added. The mixture was stirred for 2 h at 50°C then NaBH(OAc)_3_ (195 mg, 0.925 mmol) was added in one portion. The mixture was stirred at room temperature for 3 h then was partitioned between DCM (50 mL) and a sat. NaHCO_3_ (20 mL). The organic phase was dried over Na_2_SO_4_, filtered and evaporated in vacuo. The crude product was then purified by chromatography to afford 8-fluoro-2-({[trans-4-(7-fluoro-2-oxo-2,3-dihydro-1H-pyrido[2,3-b][1,4]oxazin-1-yl)cyclohexyl]amino}methyl)[1]benzopyrano[4,3-b]pyrrol-4(1H)-one (**Compound 1**). Yield = 78%.

***Synthesis of 1-(trans-4-{[(8-fluoro-4-oxo-1,4-dihydro[1]benzopyrano[4,3-b]pyrrol-2-yl)methyl]amino}cyclohexyl)-2-oxo-1,2-dihydroquinoline-7-carbonitrile hydrochloride (Compound 2) and*** ***8-fluoro-2-({[trans-4-(7-fluoro-2-oxoquinolin-1(2H)-yl)cyclohexyl]amino} methyl)[1]benzopyrano[4,3-b]pyrrol-4(1H)-one hydrochloride (Compound 6)***

The final Compounds 2 and 6 were prepared as described herein below, following the synthetic pathway C:

**Step 1**: Intermediate compounds having general formula **b** were obtained as a mixture of isomers following the same procedure described in “Synthetic pathway B, Step 2”.

*Tert-butyl [4-(5-cyano-2-iodoanilino)cyclohexyl]carbamate* (**2b**). Reagents: 3-amino-4-iodobenzonitrile (**2a**: 13 g, 53.3 mmol), *tert*-butyl (4-oxocyclohexyl)carbamate (14 g, 64 mmol), DMF (350 mL), TFA (33.8 mL, 442 mmol), NaBH(OAc)_3_ (16.9 g, 80 mmol). Yield: 24%. LC-MS [M+H]^+^ = 442.2.

*Tert-butyl [4-(5-fluoro-2-iodoanilino)cyclohexyl]carbamate* (**6b**). Reagents: 5-fluoro-2-iodoaniline (**6a**: 10 g, 42 mmol), *tert*-butyl (4-oxocyclohexyl)carbamate (10.7 g, 50.6 mmol), DMF (300 mL), TFA (26.7 mL, 349 mmol), NaBH(OAc)_3_ (13.3 g, 63 mmol). Yield: 33%. LC-MS [M+H]^+^ =435.2.

**Step 2**: The proper intermediate compound having general formula **b** (1 eq.), Pd(PtBu_3_)_3_ (0.07 eq.) and ethyl acrylate (1.2 eq.) were suspended in triethylamine. The resulting mixture was stirred for 90 min at 130°C then was cooled and partitioned between water and EtOAc. The aqueous layer was extracted twice with EtOAc, the combined organic layers were washed with brine, dried over Na_2_SO_4_ and concentrated under vacuum. The resulting crude was purified by chromatography to afford the proper intermediate with general formula **c**.

*Ethyl (2E)-3-[2-({4-[(tert-butoxycarbonyl)amino]cyclohexyl}amino)-4-cyanophenyl]prop-2-enoate* (**2c**). Reagents: *tert*-butyl [4-(5-cyano-2-iodoanilino)cyclohexyl]carbama*te* (**2b**: 5.2 g, 11.8 mmol), Pd(PtBu_3_)_3_ (0.42 g, 0.82 mmol), ethyl acrylate (1.54 mL, 14.16 mmol). Yield = 88%. LC-MS [M+H]^+^ = 414.4.

*Ethyl (2E)-3-[2-({4-[(tert-butoxycarbonyl)amino]cyclohexyl}amino)-4-fluorophenyl]prop-2-enoate* (**6c**). Reagents: *tert*-butyl [4-(5-fluoro-2-iodoanilino)cyclohexyl]carbamate (**6b**: 3.7 g, 8.52 mmol), Pd(PtBu_3_)_3_ (0.31 g, 0.60 mmol), ethyl acrylate (1.11 mL, 10.22 mmol). Yield = 83%. LC-MS [M+H]^+^ = 407.2.

**Step 3**: The proper intermediate compound having general formula **c** was dissolved in EtOAc. 10% Pd/C (0.8 eq.) was added and the mixture was stirred under hydrogen atmosphere (1 atm) overnight. The catalyst was then removed by filtration and the solvent was evaporated in vacuo to obtain intermediates **2d** or **6d** as a mixture of isomers.

*ethyl 3-[2-({4-[(tert-butoxycarbonyl)amino]cyclohexyl}amino)-4-cyanophenyl]propanoate* (**2d**). Reagents: Ethyl (2E)‐3‐{2‐[(4‐{[(tert‐butoxy)carbonyl]amino}cyclohexyl)amino]‐4‐cyanophenyl}prop‐2‐enoate (**2c**: 37.9 g, 91.7 mmol), EtOAc (379 mL) and 10% Pd/C (7.6 g). Yield = 94%, LC-MS [M+H]^+^ = 416.4

*ethyl 3-[2-({4-[(tert-butoxycarbonyl)amino]cyclohexyl}amino)-4-fluorophenyl]propanoate* (**6d**). Reagents: Ethyl (2E)-3-[2-({4-[(tert-butoxycarbonyl)amino]cyclohexyl}amino)-4-fluorophenyl]prop-2-enoate (**6c**: 2.8 g, 6.8 mmol), EtOAc (28 mL) and 10% Pd/C (564 mg). Yield = 91%, LC-MS [M+H]^+^ = 409.1.

**Step 4**: LiOH·H_2_O (1.9 eq.) was added to the proper intermediate **d** (1 eq.) previously dissolved in THF/H_2_O (3.5:1), and the mixture was stirred at room temperature overnight. The solvent was evaporated in vacuo, the residue was treated with MeCN, filtered and concentrated in vacuo. The crude title product **e** (mixture of isomers) was progressed without any further purification.

*3-[2-({4-[(tert-butoxycarbonyl)amino]cyclohexyl}amino)-4-cyanophenyl]propanoic acid* (**2e**). Reagents: ethyl 3-[2-({4-[(tert-butoxycarbonyl)amino]cyclohexyl}amino)-4-cyanophenyl] propanoate (**2d**: 43.5 g, 0.11 mol), LiOH·H_2_O (8.8 g, 0.21 mol), THF/H_2_O (455/136 mL). LC-MS [M+H]^+^ = 388.1.

*3-[2-({4-[(tert-butoxycarbonyl)amino]cyclohexyl}amino)-4-fluorophenyl]propanoic acid* (**6e**). Reagents: ethyl 3-[2-({4-[(tert-butoxycarbonyl)amino]cyclohexyl}amino)-4-fluorophenyl] propanoate (**6d**: 4.1 g, 10 mmol), LiOH·H_2_O (796 mg, 19 mmol), THF/H_2_O (50/12 mL). LC-MS [M+H]^+^ = 381.1.

**Step 5**: Intermediate compound **e** (1 eq.) was dissolved in DMF, TEA (2 eq.) was added and the mixture was cooled to 0°C. HATU (1 eq.) was added at the same temperature and the mixture was allowed to slowly reach room temperature. After stirring overnight the mixture was partitioned between water and EtOAc. The organic phase was washed with brine and dried over Na_2_SO_4_. The volatiles were removed under vacuum and the crude residue was purified by flash chromatography to obtain the *trans* diastereoisomer having general formula **f** *tert-butyl [trans-4-(7-cyano-2-oxo-3,4-dihydroquinolin-1(2H)-yl)cyclohexyl]carbamate* (**2f**). Reagents: 3-[2-({4-[(tert-butoxycarbonyl)amino]cyclohexyl} amino)-4-cyanophenyl]propanoic acid (**2e**: 42.6 g, 0.11 mol), DMF (910 mL), TEA (30.5 mL, 0.22 mol), HATU (41.6 g, 0.11 mol). Yield = 35%. LC-MS [M+H]^+^ = 370.2.

*tert-butyl [trans-4-(7-fluoro-2-oxo-3,4-dihydroquinolin-1(2H)-yl)cyclohexyl]carbamate* (**6f**). Reagents: 3-[2-({4-[(tert-butoxycarbonyl)amino]cyclohexyl}amino)-4-fluorophenyl] propanoic acid (**6e**: 3.8 g, 10 mmol), DMF (85 mL), TEA (2.8 mL, 20 mmol), HATU (3.8 g, 10 mmol). Yield = 41%. LC-MS [M+H]^+^ = 363.1.

**Step 6**: Intermediate **f** (1 eq.) was dissolved in DCE, NBS (1.3 eq.) and AIBN (0.15 eq.) were added and the mixture was stirred at 80°C for 4 h. Water was added, the organic phase was separated, washed with brine and evaporated in vacuo to obtain the crude product **g** that was progressed without any further purification.

*tert-butyl [trans-4-(7-cyano-2-oxoquinolin-1(2H)-yl)cyclohexyl]carbamate* (**2g**). Reagents: tert-butyl [trans-4-(7-cyano-2-oxo-3,4-dihydroquinolin-1(2H)-yl)cyclohexyl]carbamate (**2f**: 13.7 g 37.2 mmol), DCE (274 mL), NBS (8.6 g, 48.4 mmol), AIBN (917 mg, 5.6 mmol). LC-MS [M+H]^+^ = 368.2.

*tert-butyl [trans-4-(7-fluoro-2-oxoquinolin-1(2H)-yl)cyclohexyl]carbamate* (**6g**). Reagents: tert-butyl [trans-4-(7-fluoro-2-oxo-3,4-dihydroquinolin-1(2H)-yl)cyclohexyl]carbamate (**6f**: 1.8 g, 5 mmol), DCE (30 mL), NBS (1.15 g, 6.5 mmol), AIBN (123 mg, 0.75 mmol). LC-MS [M+H]^+^ = 361.1.

**Step 7**: Intermediate compounds having general formula **h** were prepared according to the procedure described in “Synthetic pathway B, Step 4”.

*1-(trans-4-aminocyclohexyl)-2-oxo-1,2-dihydroquinoline-7-carbonitrile.* (**2h**). Reagents: *tert-*butyl [trans-4-(7-cyano-2-oxoquinolin-1(2H)-yl)cyclohexyl]carbamate (**2g**: 10 g, 27 mmol), DCM (180 mL), TFA (25 mL). Yield = quant. LC-MS [M+H]^+^ = 268.2

*1-(trans-4-aminocyclohexyl)-7-fluoroquinolin-2(1H)-one* (**6h**). Reagents: *tert-butyl [trans-4-(7-fluoro-2-oxoquinolin-1(2H)-yl)cyclohexyl]carbamate* (**6g**: 10 g, 27.8 mmol), DCM (180 mL), TFA (25 mL). Yield = quant. LC-MS [M+H]^+^ = 261.2

**Step 8**: Final compounds were prepared according to the procedure described in “Synthesis pathway B, Step 5”.

*1-(trans-4-{[(8-fluoro-4-oxo-1,4-dihydro[1]benzopyrano[4,3-b]pyrrol-2-yl)methyl]amino} cyclohexyl)-2-oxo-1,2-dihydroquinoline-7-carbonitrile hydrochloride* (**Compound 2**). Reagents: 8-fluoro-4-oxo-1,4-dihydro[1]benzopyrano[4,3-b]pyrrole-2-carbaldehyde (**Key Int**.: 1.7 g, 4.44 mmol) and 1-(trans-4-aminocyclohexyl)-2-oxo-1,2-dihydroquinoline-7-carbonitrile (**2h**, 1 g, 3.7 mmol), acetic acid (2 drops), NaBH(OAc)_3_ (1.95 g, 9.25 mmol). Yield = 12%.

*8-fluoro-2-({[trans-4-(7-fluoro-2-oxoquinolin-1(2H)-yl)cyclohexyl]amino}methyl)[1] benzopyrano[4,3-b]pyrrol-4(1H)-one* *hydrochloride* (**Compound 6**). Reagents: 8-fluoro-4-oxo-1,4-dihydro[1]benzopyrano[4,3-b]pyrrole-2-carbaldehyde (**Key Int**.: 2.3 g, 6 mmol) and *1-(trans-4-aminocyclohexyl)-7-fluoroquinolin-2(1H)-one* (**6h**, 1.3 g, 5 mmol), acetic acid (2 drops), NaBH(OAc)_3_ (2.6 g, 12.5 mmol). Yield = 17%.

***Synthesis of 8-fluoro-2-({[trans-4-(7-fluoro-2-oxopyrido[2,3-b]pyrazin-1(2H)-yl) cyclohexyl]amino}methyl)[1]benzopyrano[4,3-b]pyrrol-4(1H)-one (formate salt) (Compound 4)***

The final Compound 4 was prepared as described hereinbelow, following the synthetic pathway D:

**Step 1**: 5-fluoro-3-nitropyridin-2-amine (1.05 g, 6.7 mmol, 1 eq.) was dissolved in DMF/MeCN 1:2 (30 mL), K_2_CO_3_ (929 mg, 6.7 mmol, 1 eq.) was added followed by ethyl bromoacetate (0.78 mL, 6.9 mmol, 1.03 eq.). The mixture was stirred at room temperature overnight then EtOAc was added followed by water. The organic phase was separated, washed with brine, dried and evaporated in vacuo. The crude material was purified by flash chromatography to obtain *N*-(5-fluoro-3-nitropyridin-2-yl)glycinate. Yield = 95%. LC-MS [M+H]^+^ = 244.2

**Step 2**: T*ert*-butyl 4-[(3-amino-6-methoxypyridin-2-yl)amino]piperidine-1-carboxylate was obtained following the same procedure described in “Synthetic pathway C, Step 3”. Reagents: *N*-(5-fluoro-3-nitropyridin-2-yl)glycinate (1.5 g, 6 mmol), EtOAc (15 mL) and 10% Pd/C (622 mg). Yield = 47%. LC-MS [M+H]^+^ = 214.0

**Step 3**: ethyl *N*-[3-({4-[(*tert*-butoxycarbonyl)amino]cyclohexyl}amino)-5-fluoropyridin-2-yl]glycinate was obtained following the same procedure described in “Synthetic pathway B, Step 2”. Reagents: T*ert*-butyl 4-[(3-amino-6-methoxypyridin-2-yl)amino]piperidine-1-carboxylate (490 mg, 2.3 mmol), *tert*-butyl (4-oxocyclohexyl)carbamate (638 mg, 3.0 mmol), DMF (30 mL), TFA (1.43 mL, 19.1 mmol), NaBH(OAc)_3_ (730 mg, 3.5 mmol). Yield = 69%. LC-MS [M+H]^+^ = 411.4.

**Step 4**: A solution of ethyl *N*-[3-({4-[(*tert*-butoxycarbonyl)amino]cyclohexyl}amino)-5-fluoropyridin-2-yl]glycinate (651 mg, 1.6 mmol) and cat. acetic acid (0.13 mL) in toluene (25 mL) was refluxed overnight. The reaction mixture was concentrated, the residue was dissolved in DCM (20 mL) and treated with MnO_2_ (2.6 g, 30 mmol, 19 eq.) at room temperature for 2 h. The solid was filtered and the solvent was evaporated in vacuo. The crude material was purified by flash chromatography to obtain *tert*-butyl [4-(7-fluoro-2-oxopyrido[2,3-b]pyrazin-1(2H)-yl)cyclohexyl]carbamate. Yield = 43%. LC-MS [M+H]^+^ = 363.4.

**Step 5**: 1-(4-aminocyclohexyl)-7-fluoropyrido[2,3-b]pyrazin-2(1H)-one was prepared according to the procedure described in “Synthetic pathway B, Step 4”. Reagents: *tert*-butyl [4-(7-fluoro-2-oxopyrido[2,3-b]pyrazin-1(2H)-yl)cyclohexyl]carbamate (217 mg, 0.6 mmol) was dissolved in DCM (5 mL), TFA (1 mL). Yield = 94%. LC-MS [M+H]^+^ = 263.1.

**Step 6**: The final **Compound 4**, 8-fluoro-2-({[*trans*-4-(7-fluoro-2-oxopyrido[2,3-b]pyrazin-1(2H)-yl)cyclohexyl]amino}methyl)[1]benzopyrano[4,3-b]pyrrol-4(1H)-one (formate salt), was prepared according to the procedure described in “Synthesis pathway B, Step 5”. Reagents: 8-fluoro-4-oxo-1,4-dihydro[1]benzopyrano[4,3-b]pyrrole-2-carbaldehyde (**Key Int**.: 236 mg, 0.61 mmol), 1-(4-aminocyclohexyl)-7-fluoropyrido[2,3-b]pyrazin-2(1H)-one (134 mg, 0.51 mmol), DCM (20 mL), acetic acid (2 drops), NaBH(OAc)_3_ (270 mg, 1.27 mmol). Yield = 10%. ***Synthesis of 8-fluoro-2-({[trans-4-(7-methoxy-2-oxopyrido[2,3-b]pyrazin-1(2H)-yl) cyclohexyl]amino}methyl)[1]benzopyrano[4,3-b]pyrrol-4(1H)-one hydrochloride (Compound 5)***

The final Compound 5 was prepared as described hereinbelow, following the synthetic pathway E:

**Step 1**: 200 mg (0.55 mmol) of *tert*-butyl [4-(7-fluoro-2-oxopyrido[2,3-b]pyrazin-1(2H)-yl)cyclohexyl]carbamate (**INT 1**), prepared as previously described (Synthetic pathway D, Step 5), was dissolved in MeOH (10 mL). NaOMe (25% wt solution in MeOH, 25 mL) was added and the mixture was stirred at 50°C for 3 h. The solvent was evaporated in vacuo, DCM was added and the solution was washed with sat. NaHCO_3_. The organic phase was separated, dried over Na_2_SO_4_ and concentrated in vacuo to obtain *tert*-butyl [4-(7-methoxy-2-oxopyrido[2,3-b]pyrazin-1(2H)-yl)cyclohexyl]carbamate, that was progressed without any further purification. LC-MS [M+H]^+^ = 375.4.

**Step 2**: 1-(4-aminocyclohexyl)-7-methoxypyrido[2,3-b]pyrazin-2(1H)-one was prepared according to the procedure described in “Synthetic pathway B, Step 4”. Reagents: *tert*-butyl [4-(7-methoxy-2-oxopyrido[2,3-b]pyrazin-1(2H)-yl)cyclohexyl]carbamate (198 mg, 0.53 mmol), DCM (5 mL), TFA (1 mL). Yield = 82%. LC-MS [M+H]^+^ = 275.2.

**Step 3**: The final **Compound 5**, 8-fluoro-2-({[trans-4-(7-methoxy-2-oxopyrido[2,3-b]pyrazin-1(2H)-yl)cyclohexyl]amino}methyl)[1]benzopyrano[4,3-b]pyrrol-4(1H)-one hydrochloride, was prepared according to the procedure described in “Synthetic pathway B, Step 5”. Reagents: 8-fluoro-4-oxo-1,4-dihydro[1]benzopyrano[4,3-b]pyrrole-2-carbaldehyde (**Key Int**.: 194 mg, 0.50 mmol), 1-(4-aminocyclohexyl)-7-methoxypyrido[2,3-b]pyrazin-2(1H)-one (115 mg, 0.42 mmol), DCM (20 mL), acetic acid (2 drops), NaBH(OAc)_3_ (221 mg, 1.05 mmol). Yield = 43%.

***Synthesis of 8-fluoro-2-({[trans-4-(7-fluoro-2-oxo-1,5-naphthyridin-1(2H)-yl) cyclohexyl]amino}methyl)[1]benzopyrano[4,3-b]pyrrol-4(1H)-one hydrochloride (Compound 3)***

The final Compound 3 was prepared as described hereinbelow, following the synthetic pathway F:

**Step 1**: *tert*-butyl {4-[(2-bromo-5-fluoropyridin-3-yl)amino]cyclohexyl}carbamate was obtained following the same procedure described in “Synthetic pathway B, Step 2”. Reagents: 2-bromo-5-fluoropyridin-3-amine (4.5 g, 23.5 mmol), *tert*-butyl (4-oxocyclohexyl)carbamate (6.5 g, 30.5 mmol), DMF (250 mL), NaBH(OAc)_3_ (6.86 g, 32.3 mmol, 1.5 eq.). Yield = 98%. LC-MS [M+H]^+^ = 388.2.

**Step 2**: ethyl (2E)-3-[3-({4-[(*tert*-butoxycarbonyl)amino]cyclohexyl}amino)-5-fluoropyridin-2-yl]prop-2-enoate was obtained following the same procedure described in “Synthetic pathway C, Step 2”. Reagents: *tert*-butyl {4-[(2-bromo-5-fluoropyridin-3-yl)amino]cyclohexyl}carbamate (7.7 g, 20 mmol), Pd(PtBu_3_)_3_ (0.61 g, 1.2 mmol), ethyl acrylate (2.61 mL, 24 mmol). Yield = 66%. LC-MS [M+H]^+^ = 408.5.

**Step 3**: ethyl 3-[3-({4-[(*tert*-butoxycarbonyl)amino]cyclohexyl}amino)-5-fluoropyridin-2-yl]propanoate was obtained following the same procedure described in “Synthetic pathway C, Step 3”. Reagents: ethyl (2E)-3-[3-({4-[(*tert*-butoxycarbonyl)amino]cyclohexyl}amino)-5-fluoropyridin-2-yl]prop-2-enoate (4.9 g, 12 mmol), EtOAc (49 mL) and 10% Pd/C (1.02 g, 9.6 mmol). Yield = 97%. LC-MS [M+H]^+^ = 410.5.

**Step 4**: 3-[3-({4-[(*tert*-butoxycarbonyl)amino]cyclohexyl}amino)-5-fluoropyridin-2-yl] propanoate (1.8 g, 4.5 mmol) was dissolved in THF (20 mL), *t*-BuONa (0.87g, 9 mmol) was added and the mixture was stirred at 50°C for 4 h. EtOAc was added followed by water. The organic phase was separated, dried over Na_2_SO_4_ and evaporated in vacuo**.** The crude was purified by Si-column (cy to cy/EtOAc 1:1) to obtain the diastereoisomer *tert*-butyl [trans-4-(7-fluoro-2-oxo-3,4-dihydro-1,5-naphthyridin-1(2H)-yl)cyclohexyl]carbamate. Yield = 11%. LC-MS [M+H]^+^ = 364.4.

**Step 5**: *tert*-butyl [trans-4-(7-fluoro-2-oxo-1,5-naphthyridin-1(2H)-yl)cyclohexyl]carbamate was obtained following the same procedure described in “Synthetic pathway C, Step 6”. Reagents: *tert*-butyl [trans-4-(7-fluoro-2-oxo-3,4-dihydro-1,5-naphthyridin-1(2H)-yl)cyclohexyl]carbamate (128 mg, 0.35 mmol), NBS (81 mg, 0.46 mmol), AIBN (11.5 mg, 0.07 mmol). Yield = 96%. LC-MS [M+H]^+^ = 362.2.

**Step 6**: 1-(trans-4-aminocyclohexyl)-7-fluoro-1,5-naphthyridin-2(1H)-one was prepared according to the procedure described in “Synthetic pathway B, Step 4”. Reagents: *tert*-butyl [trans-4-(7-fluoro-2-oxo-1,5-naphthyridin-1(2H)-yl)cyclohexyl]carbamate (121 mg, 0.30 mmol), DCM (5 mL), TFA (1 mL). Yield = 98%. LC-MS [M+H]^+^ = 262.2.

**Step 7**: 8-fluoro-2-({[trans-4-(7-fluoro-2-oxo-1,5-naphthyridin-1(2H)-yl)cyclohexyl]amino} methyl)[1]benzopyrano[4,3-b]pyrrol-4(1H)-one hydrochloride (**Compound 3**) was prepared according to the procedure described in “Synthetic pathway B, Step 5”. Reagents: 1-(trans-4-aminocyclohexyl)-7-fluoro-1,5-naphthyridin-2(1H)-one (68 mg, 0.26 mmol), 8-fluoro-4-oxo-1,4-dihydro[1]benzopyrano[4,3-b]pyrrole-2-carbaldehyde (**Key Int**.: 120 mg, 0.31 mmol), acetic acid (2 drops), NaBH(OAc)_3_ (137 mg, 0.65 mmol). Yield = 18%. ml

***Abbreviations***

AIBN: 2,2′-azobis(2-methylpropionitrile);

DCE: 1,2-dichloroethane;

DCM: dichloromethane;

DMF: dimethylformamide;

DMSO: dimethyl sulfoxide;

EtOAc: ethyl acetate;

EtOH: ethanol;

H_2_O: water;

HATU: 1-[bis(dimethylamino)methylene]-1H-1,2,3-triazolo[4,5-b]pyridinium 3-oxid hexafluorophosphate;

HCl: hydrogen chloride;

K_2_CO_3_: potassium carbonate;

LiAlH_4_: lithium aluminum hydride;

LiOH·H_2_O: lithium hydroxide monohydrate;

MeCN: acetonitrile;

MeOH: methanol;

MnO_2_: manganese(IV) oxide;

Na_2_SO_4_: sodium sulfate;

NaBH(OAc)_3_: sodium triacetoxyborohydride;

NaHCO_3_: sodium bicarbonate;

NaOMe: sodium methoxide solution

NBS: *N*-bromosuccinimide;

Pd(PtBu_3_)_3_: bis(tri-tert-butylphosphine)palladium(0);

Pd/C: palladium on carbon;

POCl_3_: phosphoryl chloride;

r.t.: room temperature;

TEA: triethylamine;

TFA: trifluoroacetic acid;

THF: tetrahydrofuran;

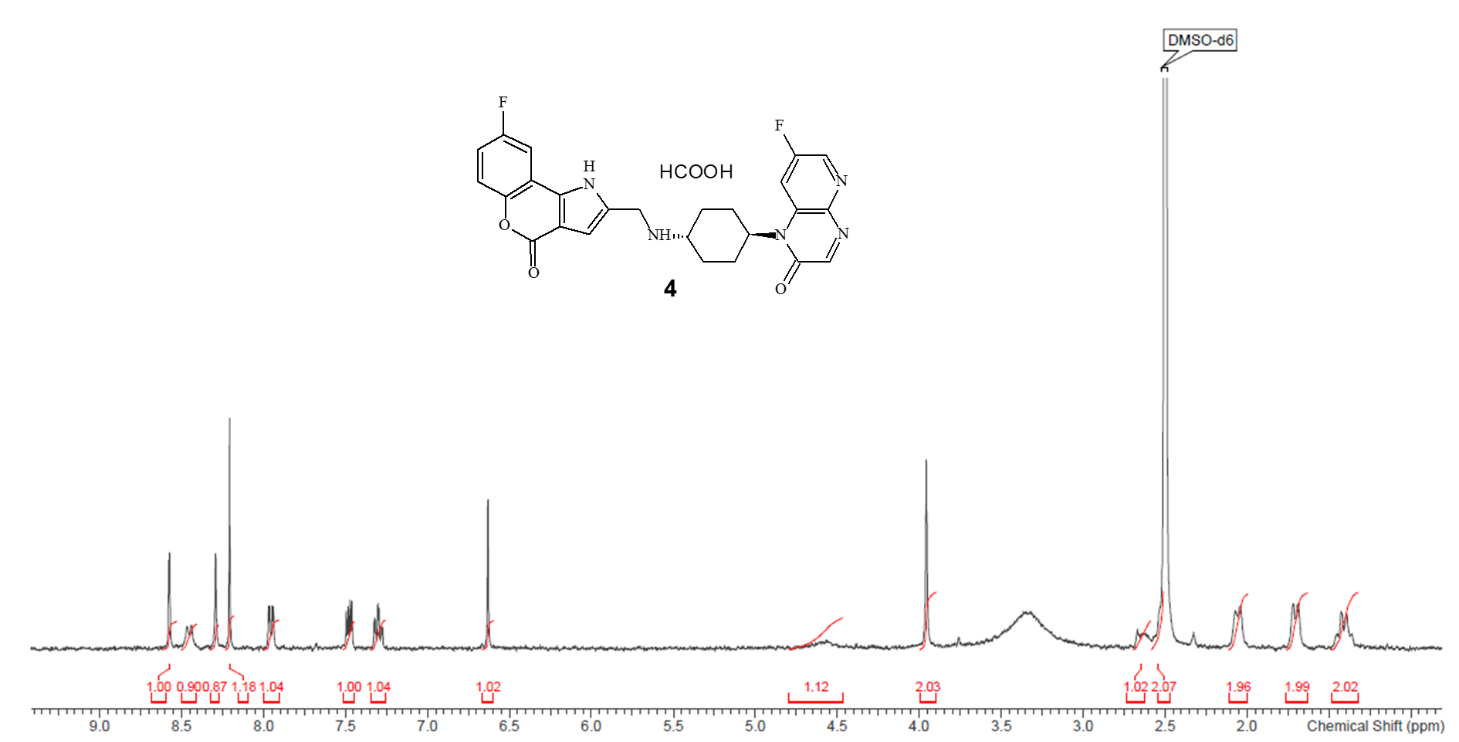


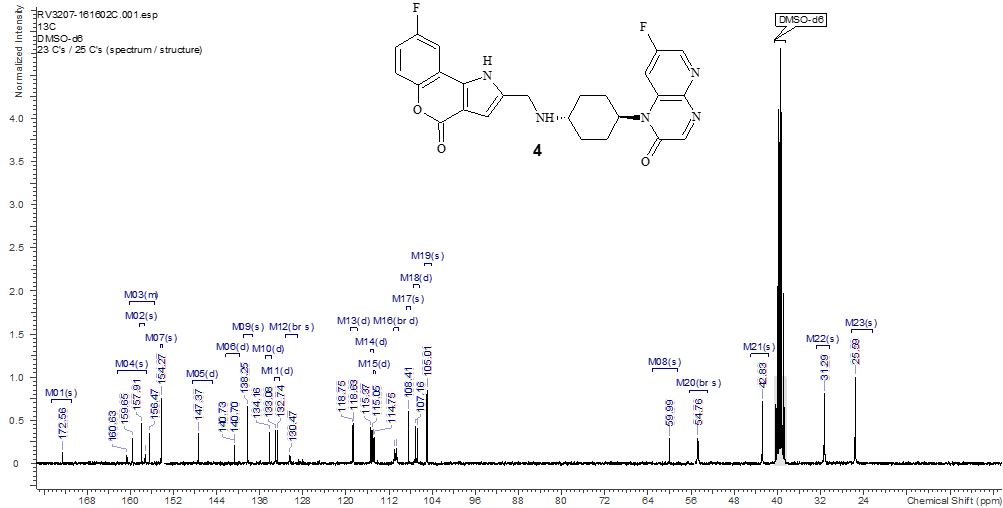


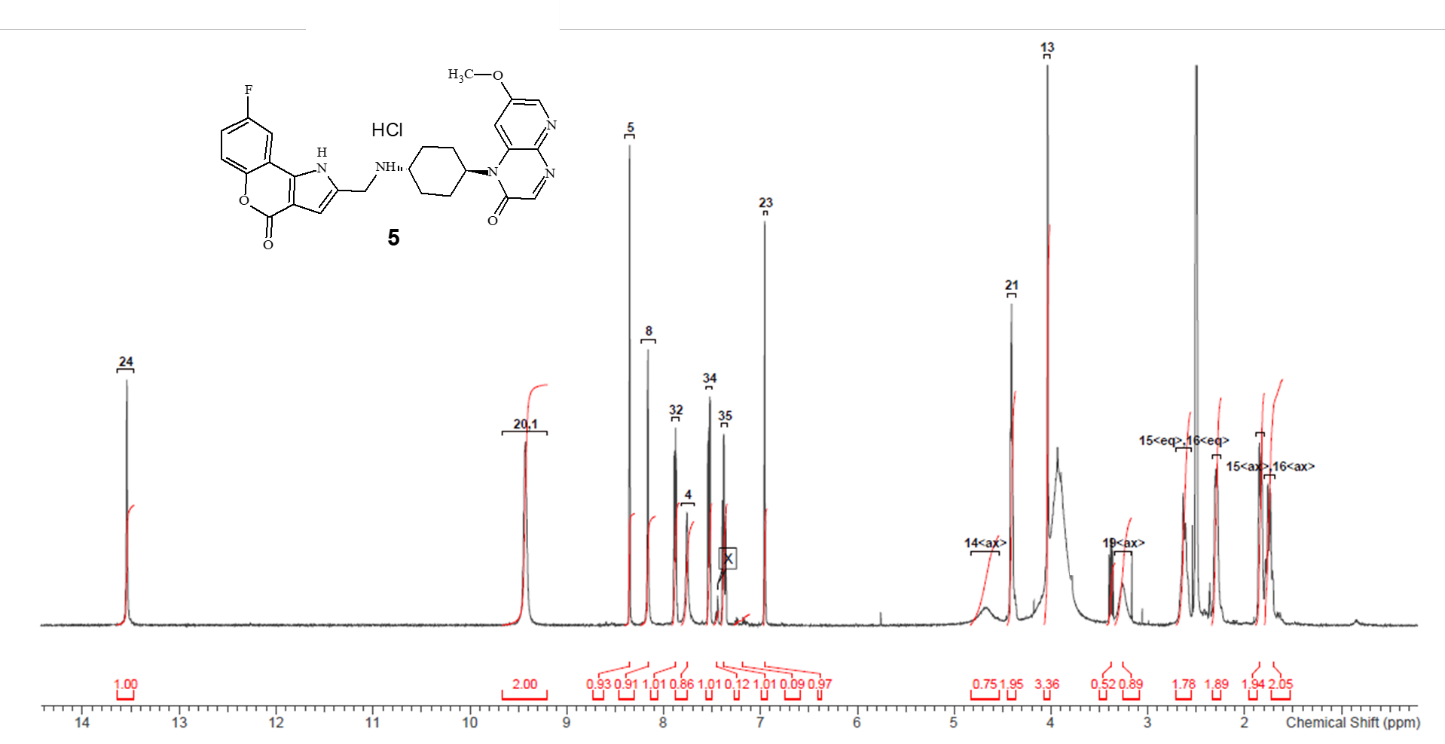

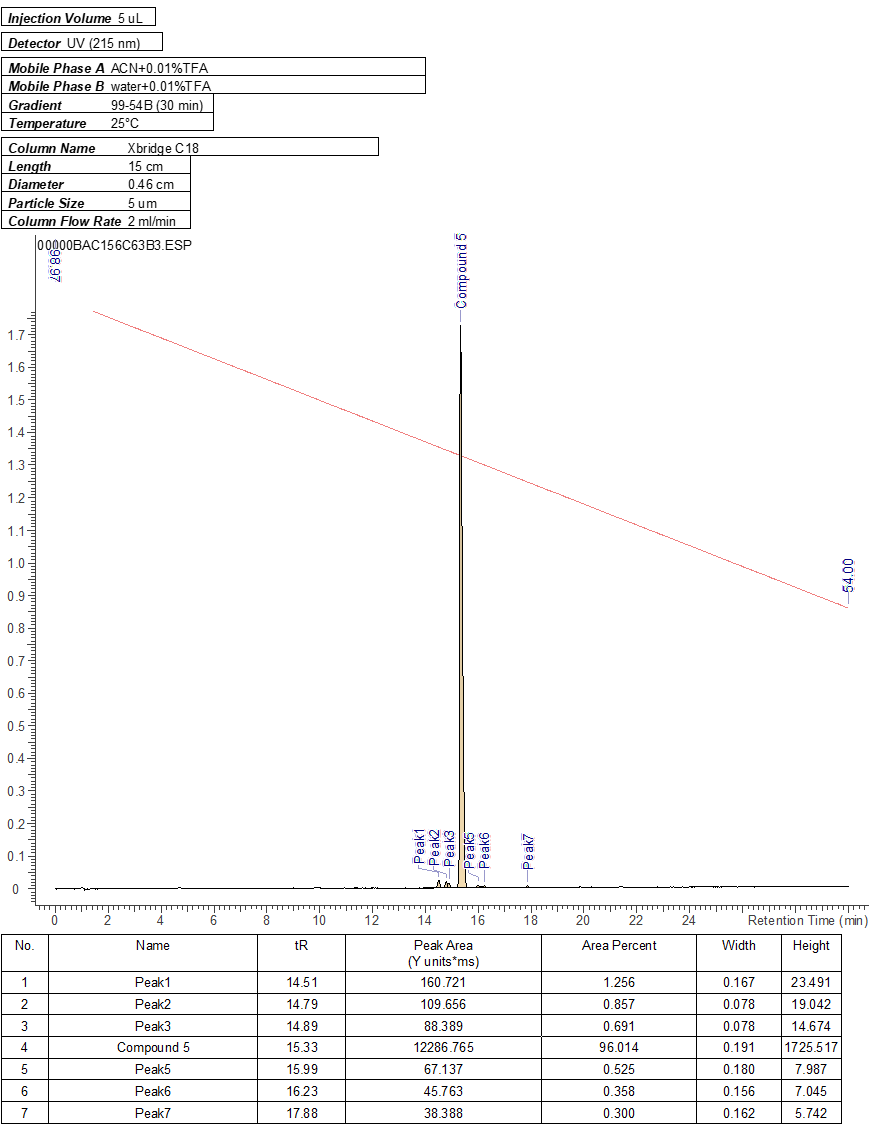

Supplement: S1 File — (DOCX) [file pone.0228509.s001.docx]
