# Supplementary material for: Antibacterial activity of novel dual bacterial DNA type II topoisomerase inhibitors
Source: PLoS One. 2020 Feb 19;15(2):e0228509. doi: 10.1371/journal.pone.0228509 (PMC7029851; doi:10.1371/journal.pone.0228509)
Supplement: S2 File — (PDF) [file pone.0228509.s002.pdf]

Original figures of enzyme Gel assays

Below are reported the original gel images underlying the results reported in table 1.

The fluorescence intensity of each band was analysed by image data acquisition software and was expressed as volume (volume of the uncalibrated quantity of band in the image after subtraction of the background intensity). Each band intensity was compared, as percentage, to vehicle sample band intensity, which served as positive control, on the same gel.

Concentration-response curves of percent inhibition at each concentration were obtained for each replicate experiment by non-linear regression analysis. Average IC_{50} and SD (standard deviation) were then calculated.

E. coli **gyrase Supercoiling**

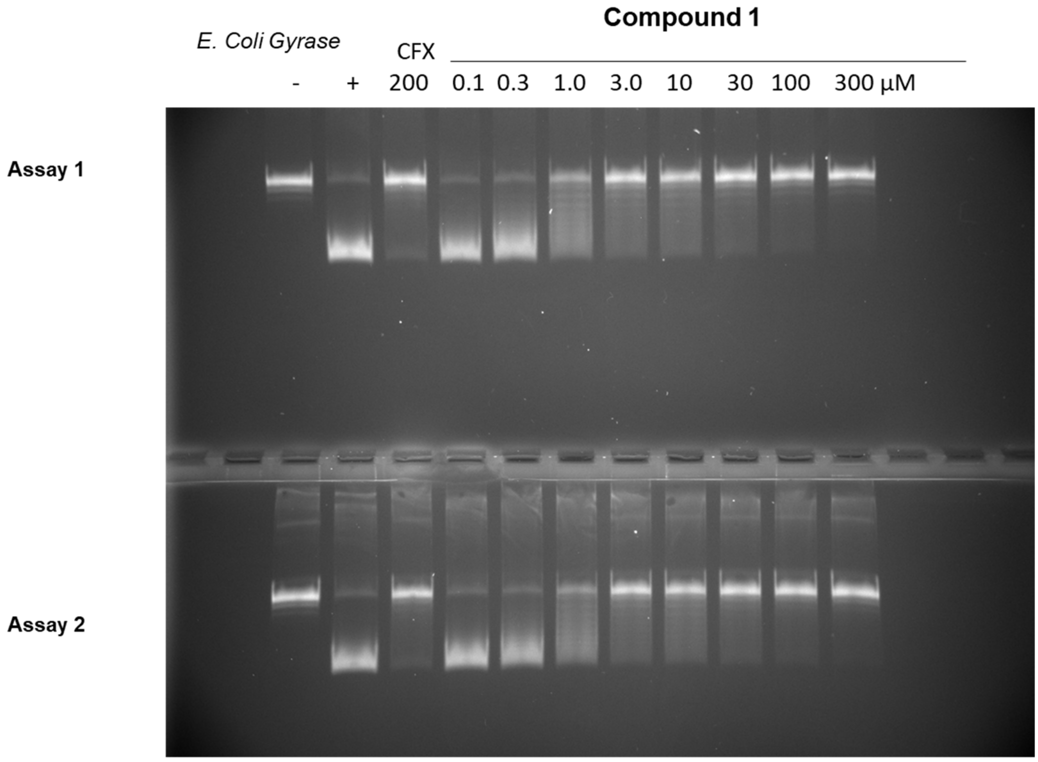

CFX= ciprofloxacin

	Assay 1	Assay 2	Average IC_{50}(µM)	Average SD
compound 1	0.34	0.16	0.25	0.13

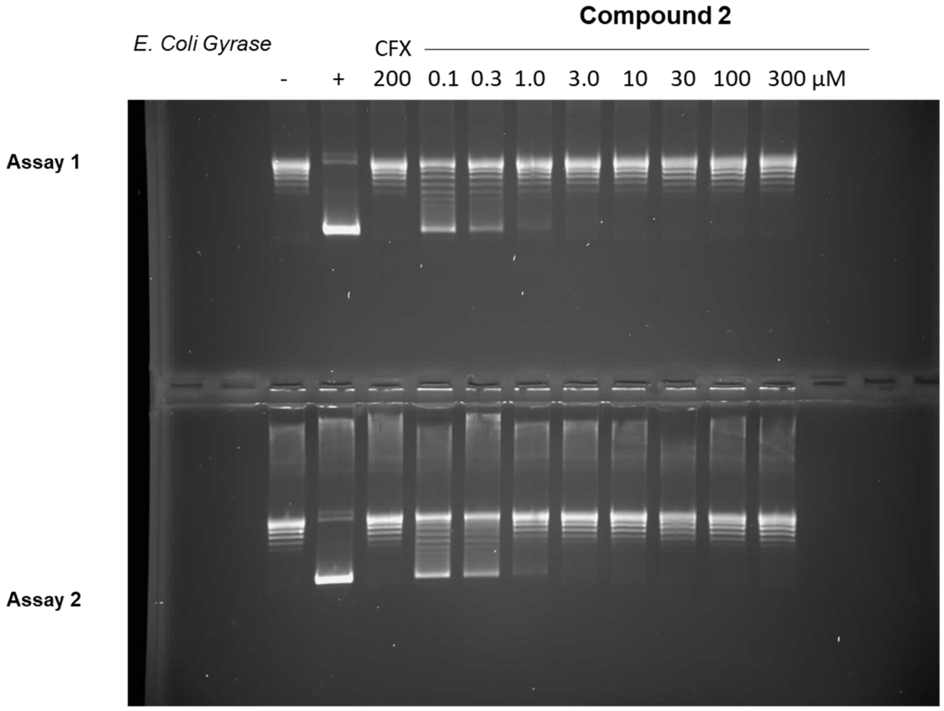

CFX= ciprofloxacin

	Assay 1	Assay 2	Average IC$_{50}$ (µM)	Average SD
compound 2	0.04	0.05	0.05	0.007

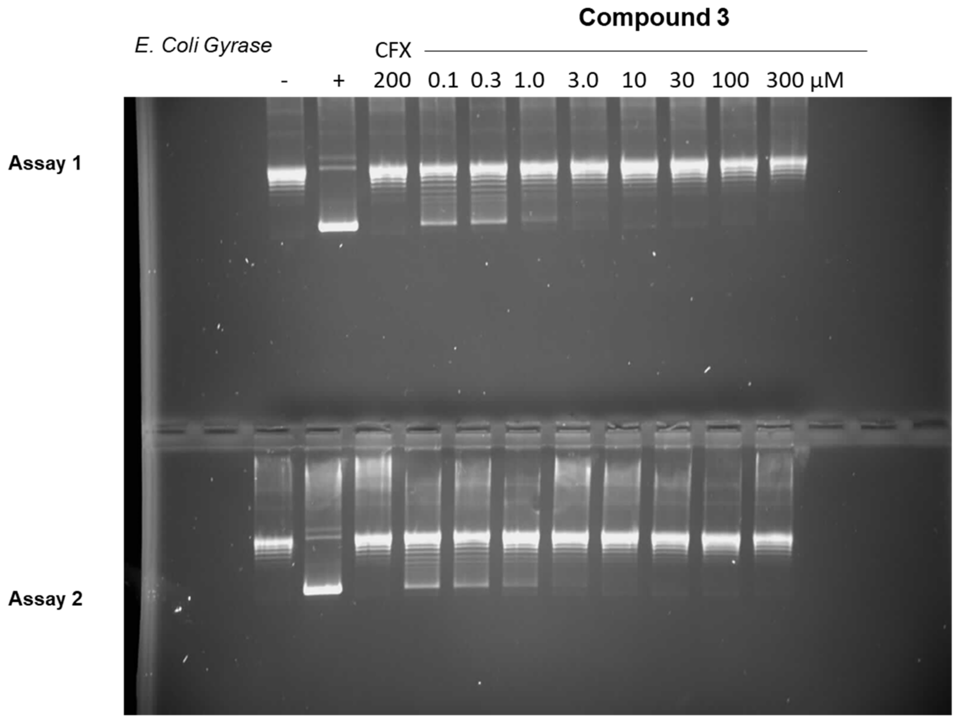

CFX= ciprofloxacin

	Assay 1	Assay 2	Average IC$_{50}$ (µM)	Average SD
compound 3	<0.1	<0.1	<0.1	<0.1

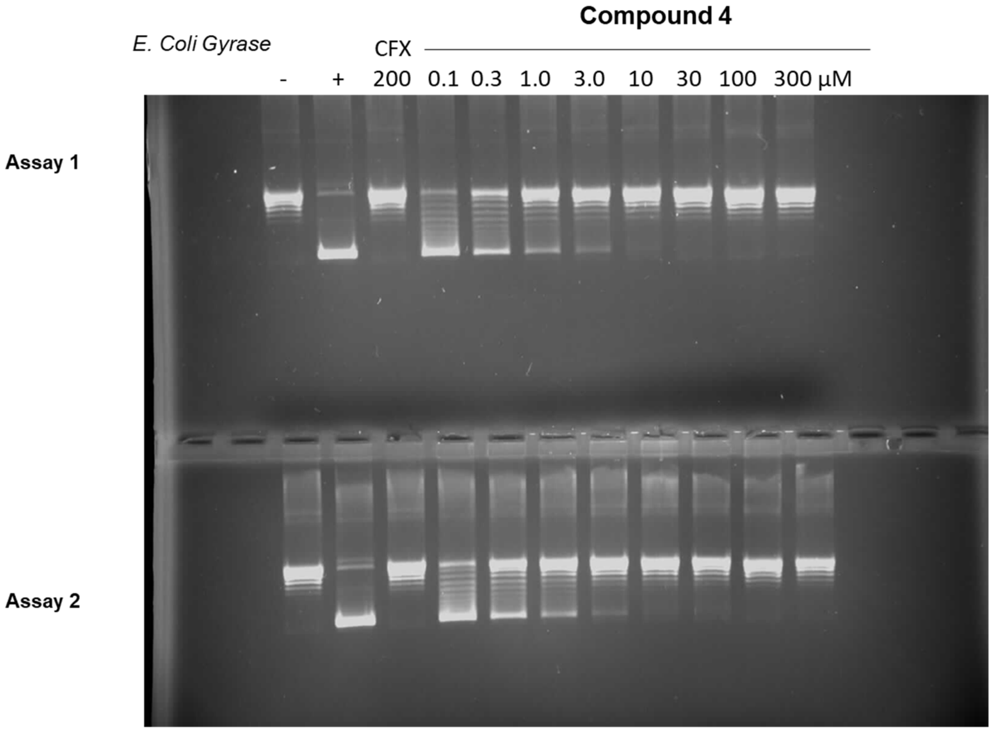

CFX= ciprofloxacin

	Assay 1	Assay 2	Average IC_{50} (µM)	Average SD
compound 4	0.15	0.12	0.13	0.02

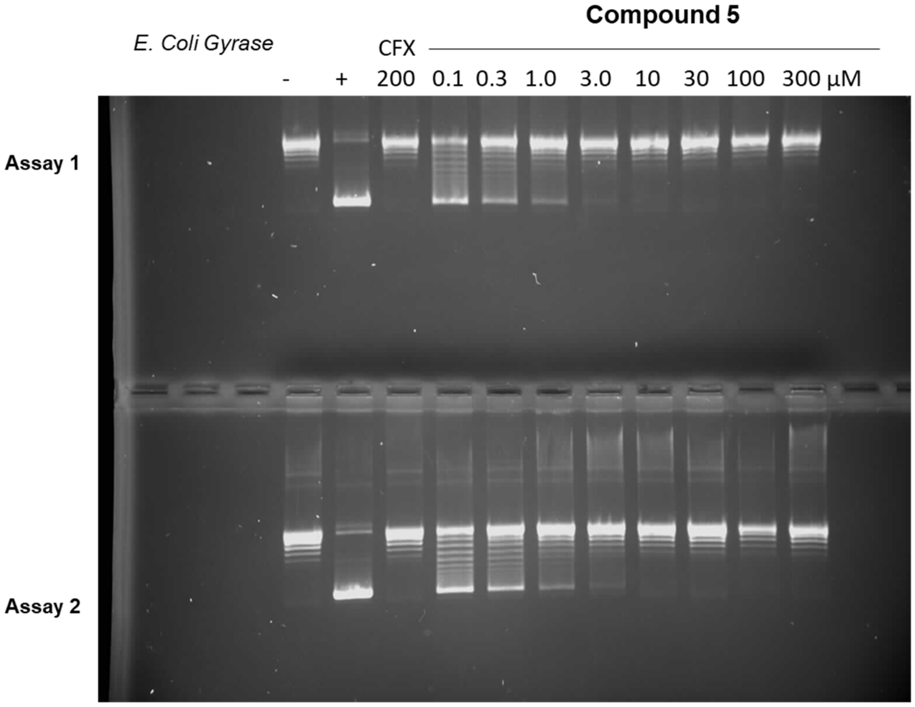

CFX= ciprofloxacin

	Assay 1	Assay 2	Average IC_{50} (µM)	Average SD
compound 5	0.09	0.1	0.1	0.007

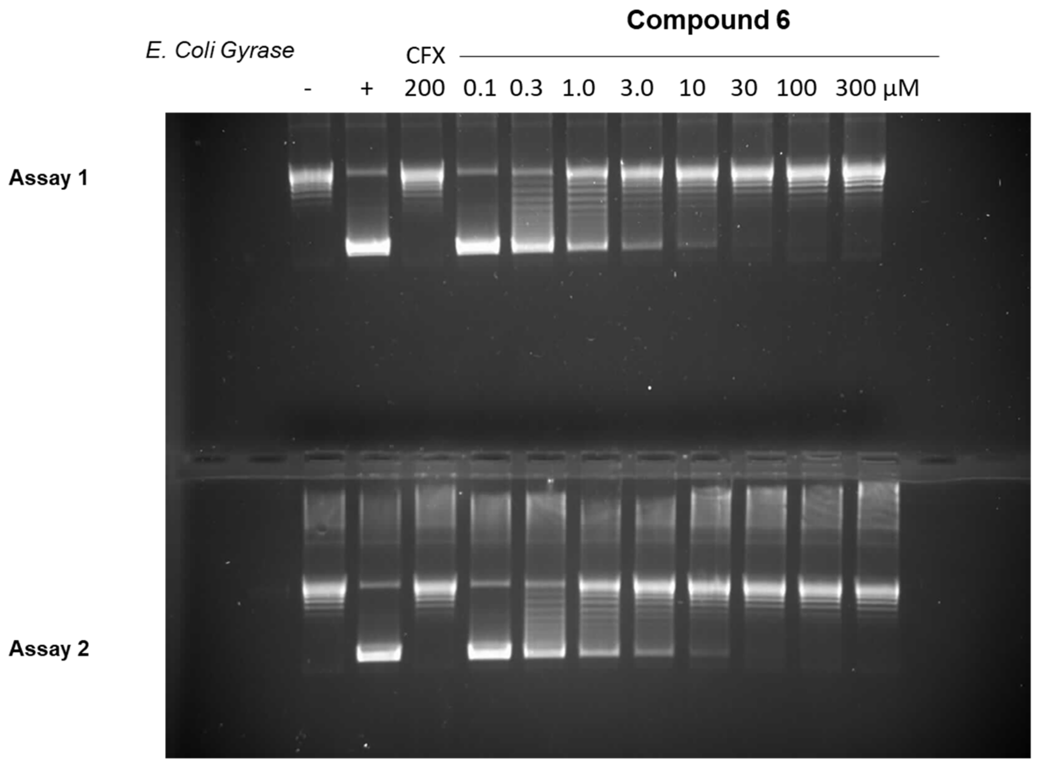

CFX= ciprofloxacin

	Assay 1	Assay 2	Average IC_{50} (µM)	Average SD
compound 6	0.13	0.12	0.13	0.007

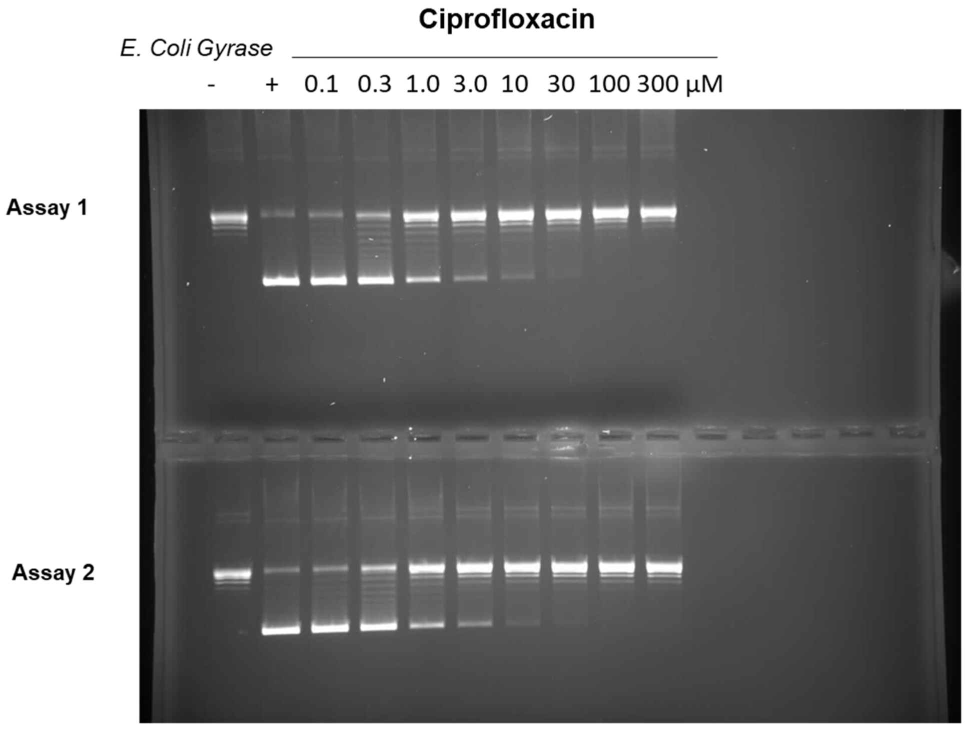

	Assay 1	Assay 2	Average IC_{50} (µM)	Average SD
ciprofloxacin	0.78	0.97	0.87	0.134

E. coli Topoisomerase IV decatenation

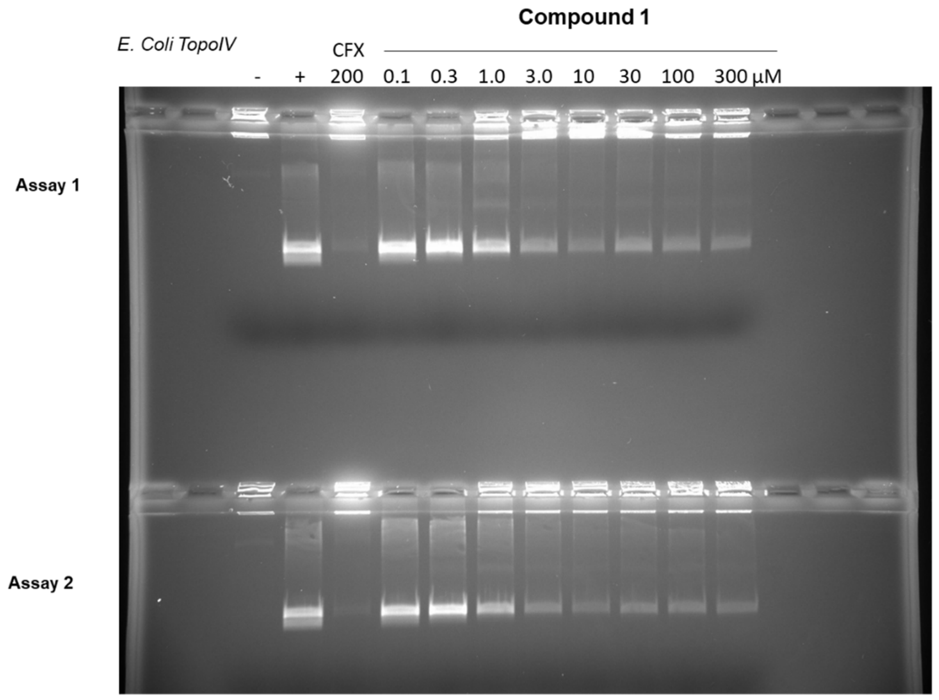

CFX= ciprofloxacin

	Assay 1	Assay 2	Average IC$_{50}$ (µM)	Average SD
compound 1	1.35	1.42	1.38	0.05

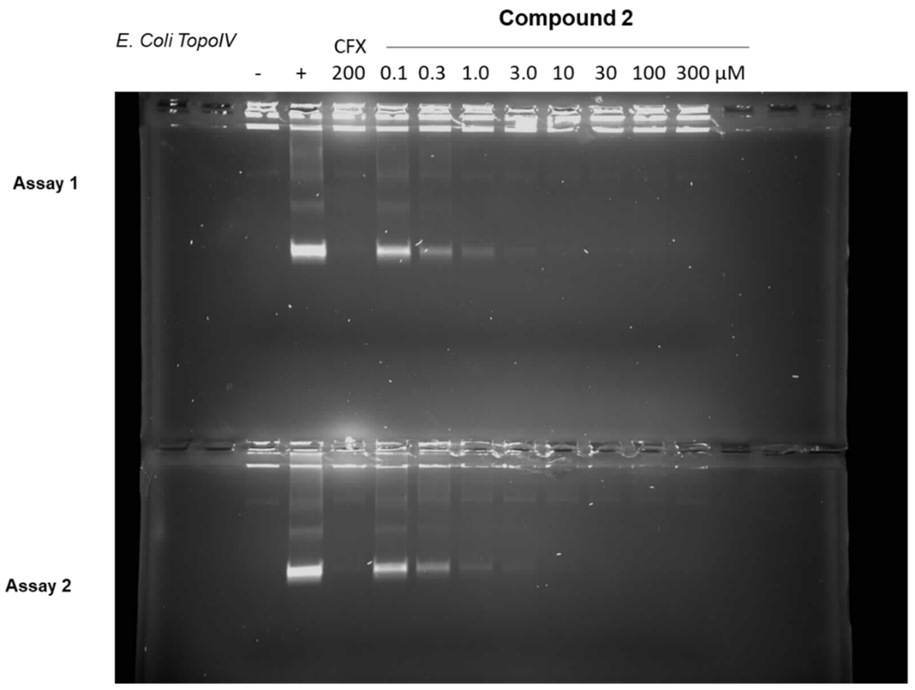

CFX= ciprofloxacin

	Assay 1	Assay 2	Average IC$_{50}$ (µM)	Average SD
compound 2	0.1	0.16	0.13	0.04

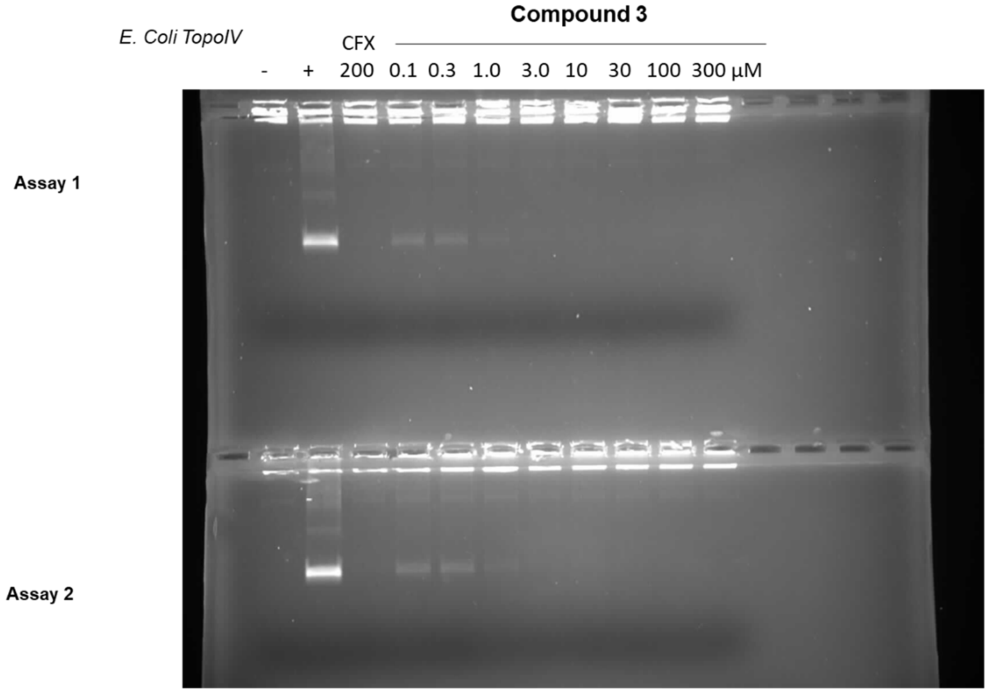

CFX= ciprofloxacin

	Assay 1	Assay 2	Average IC_{50} (µM)	Average SD
compound 3	<0.1	<0.1	<0.1	<0.1

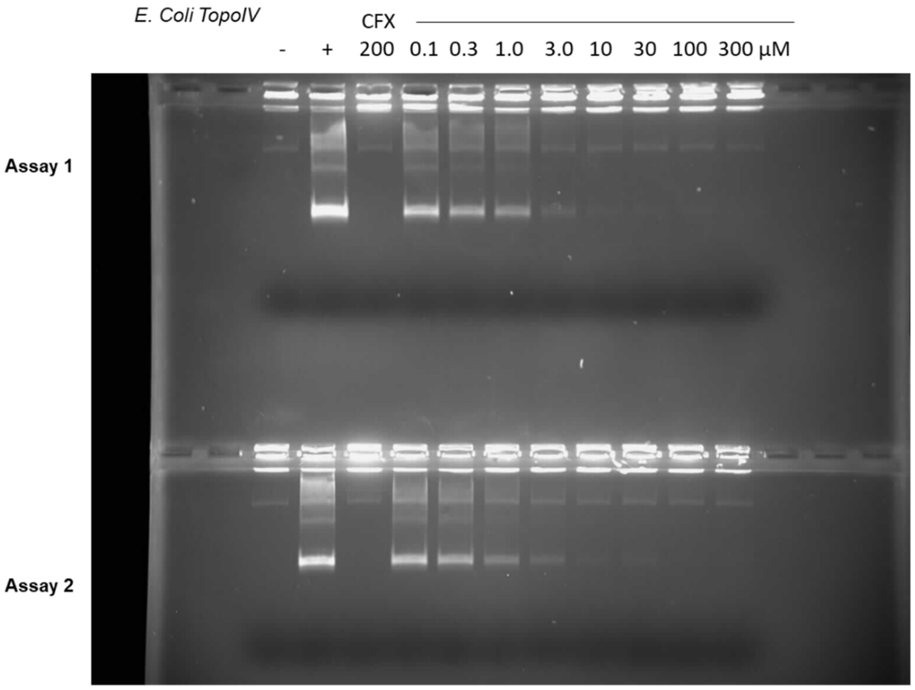

CFX= ciprofloxacin

	Assay 1	Assay 2	Average IC_{50} (µM)	Average SD
compound 4	0.5	0.2	0.3	0.2

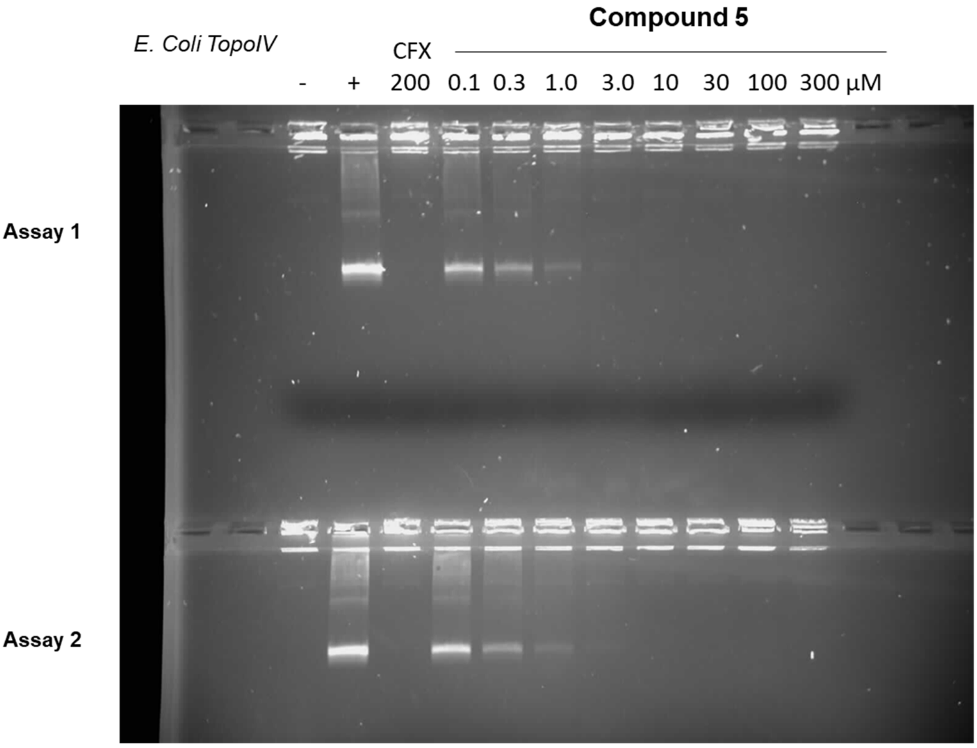

CFX= ciprofloxacin

	Assay 1	Assay 2	Average IC$_{50}$ (µM)	Average SD
compound 5	0.12	0.16	0.14	0.028

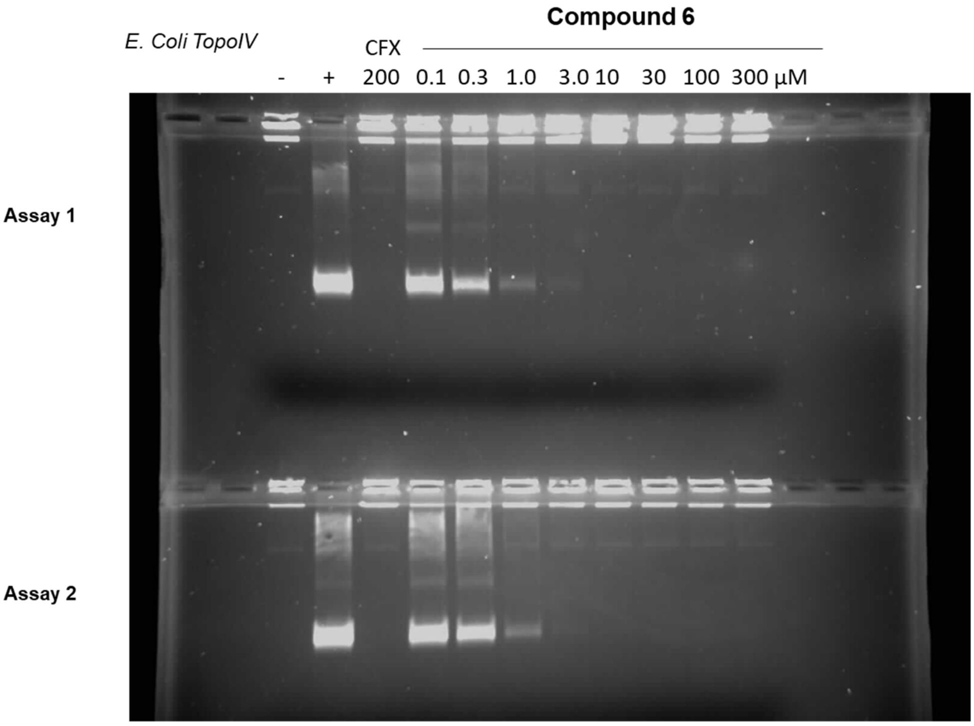

CFX= ciprofloxacin

	Assay 1	Assay 2	Average IC$_{50}$ (µM)	Average SD
compound 6	0.27	0.22	0.25	0.03

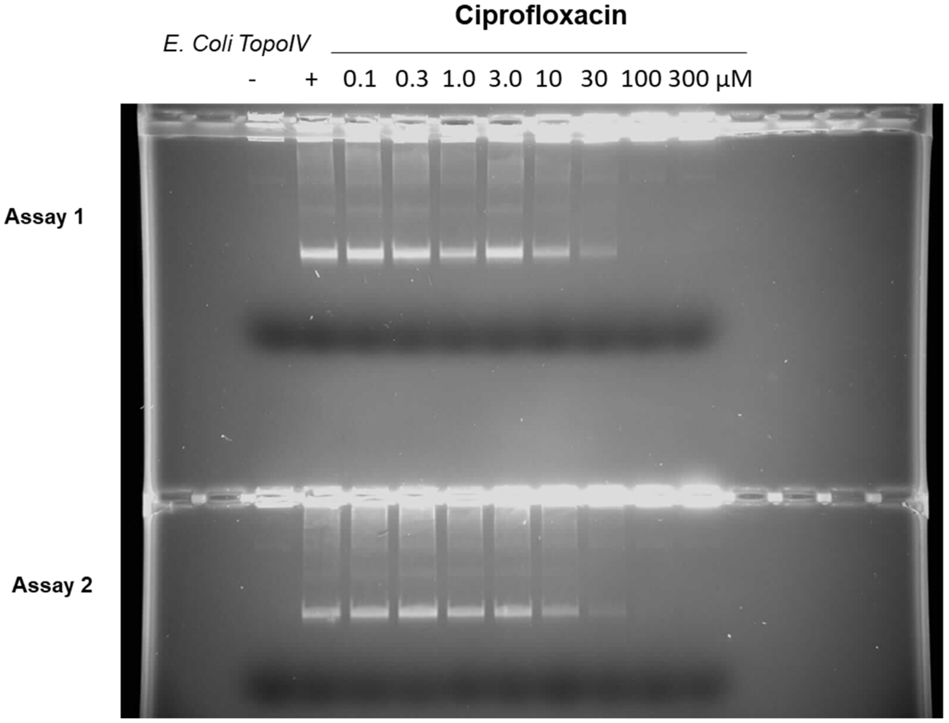

	Assay 1	Assay 2	Average IC_{50} (µM)	Average SD
ciprofloxacin	11.78	11.17	11.47	0.4

S.aureus **gyrase Supercoiling**

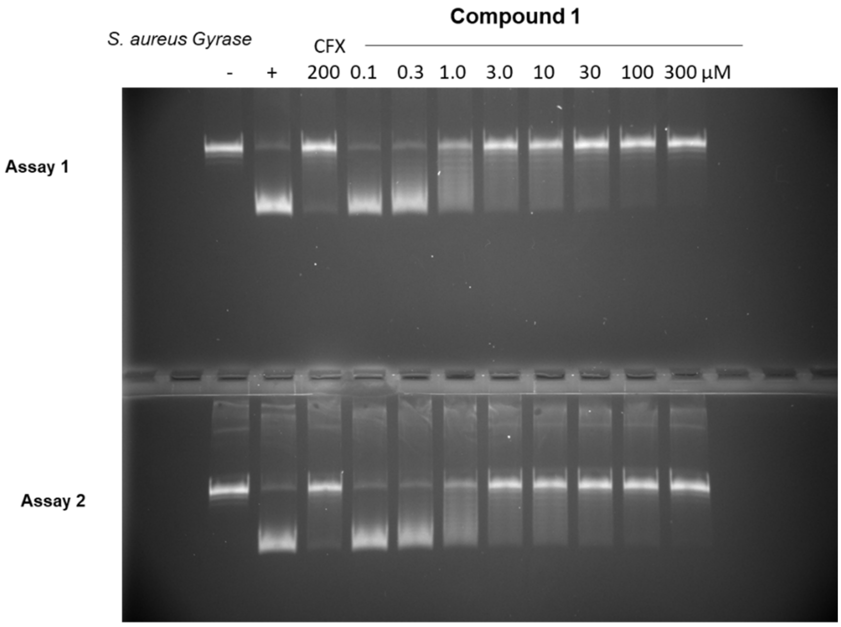

CFX= ciprofloxacin

	Assay 1	Assay 2	Average IC_{50} (µM)	Average SD
compound 1	0.25	0.31	0.28	0.04

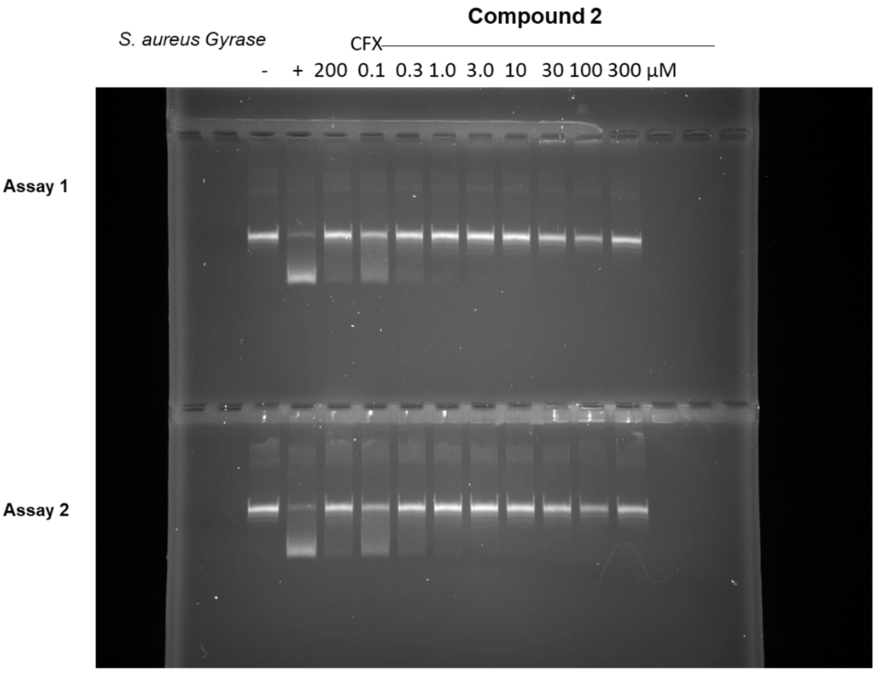

CFX= ciprofloxacin

	Assay 1	Assay 2	Average IC_{50} (µM)	Average SD
compound 2	<0.1	<0.1	<0.1	<0.1

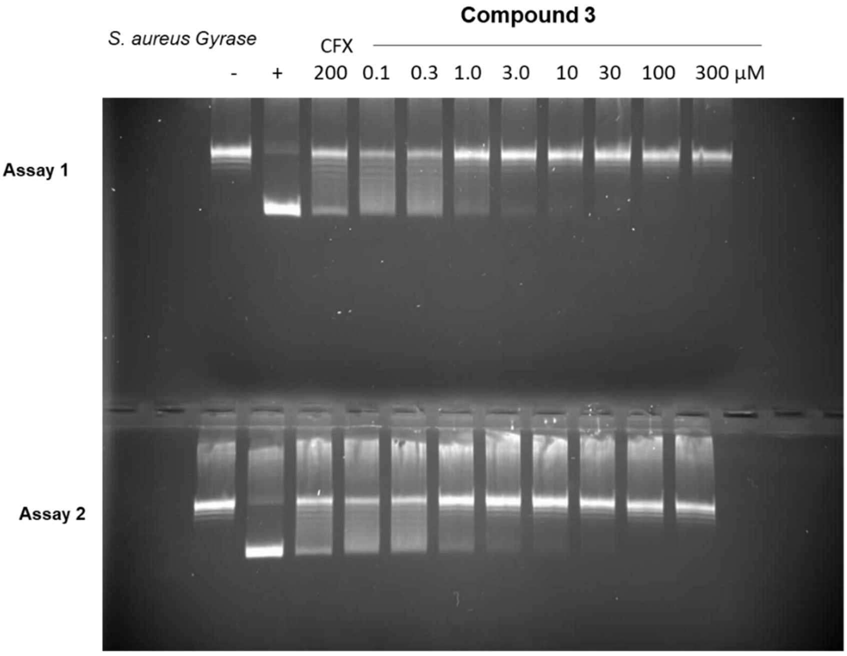

CFX= ciprofloxacin

	Assay 1	Assay 2	Average IC_{50} (µM)	Average SD
compound 3	<0.1	<0.1	<0.1	<0.1

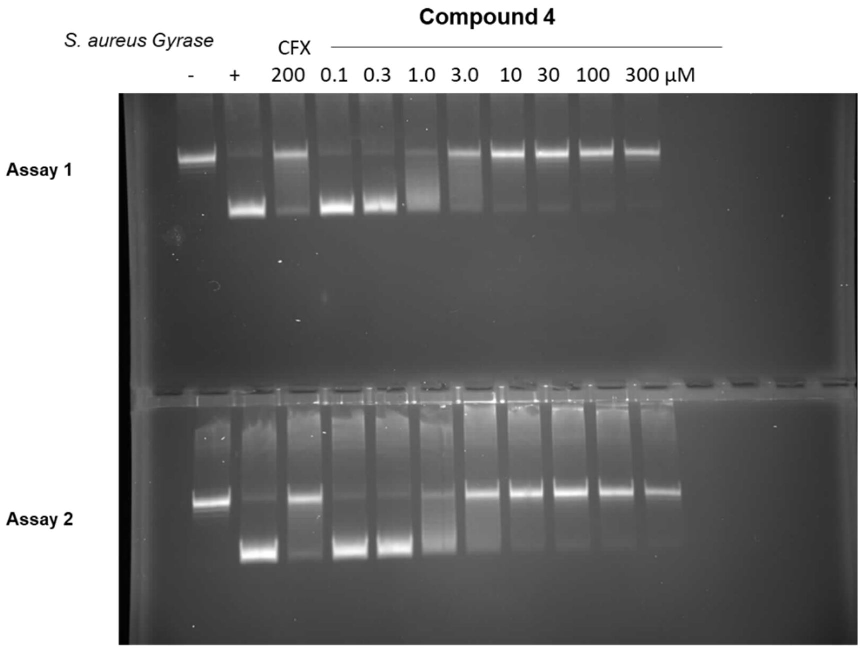

CFX= ciprofloxacin

	Assay 1	Assay 2	Average IC$_{50}$ (μM)	Average SD
compound 4	0.8	1.2	1	0.28

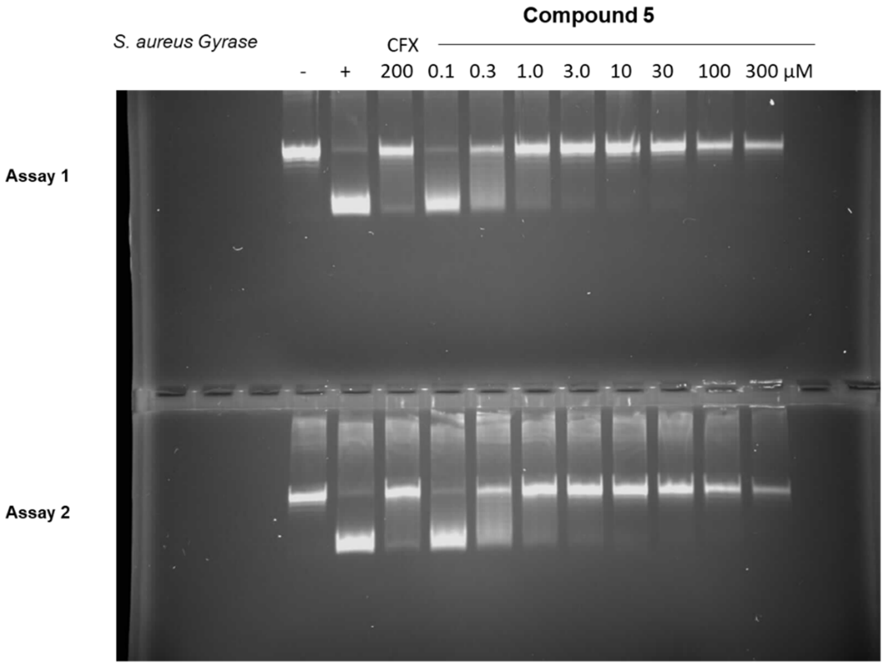

CFX= ciprofloxacin

	Assay 1	Assay 2	Average IC$_{50}$ (μM)	Average SD
compound 5	0.16	0.17	0.17	0.007

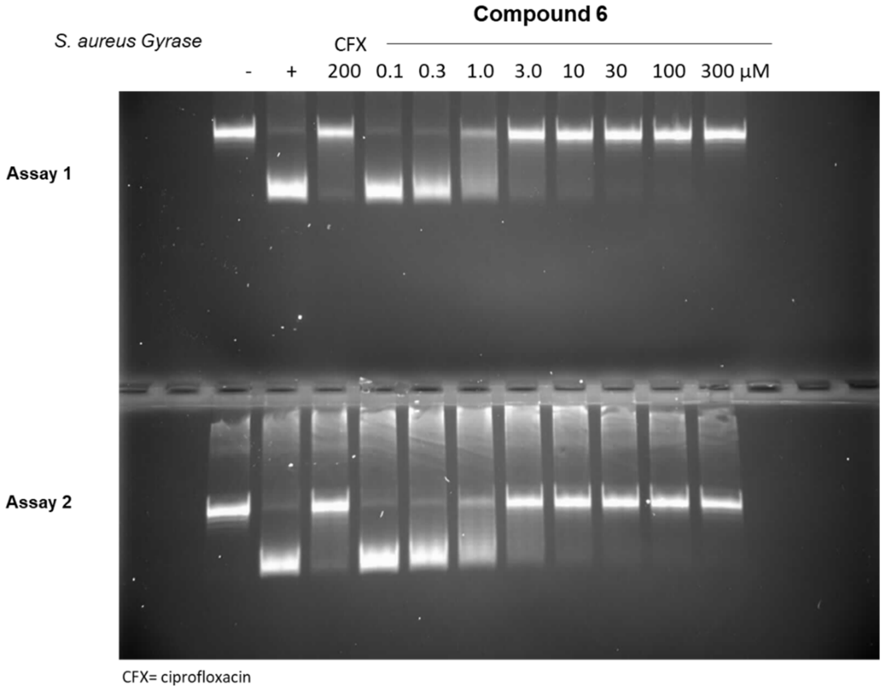

	Assay 1	Assay 2	Average IC_{50} (µM)	Average SD
compound 6	0.27	0.35	0.31	0.06

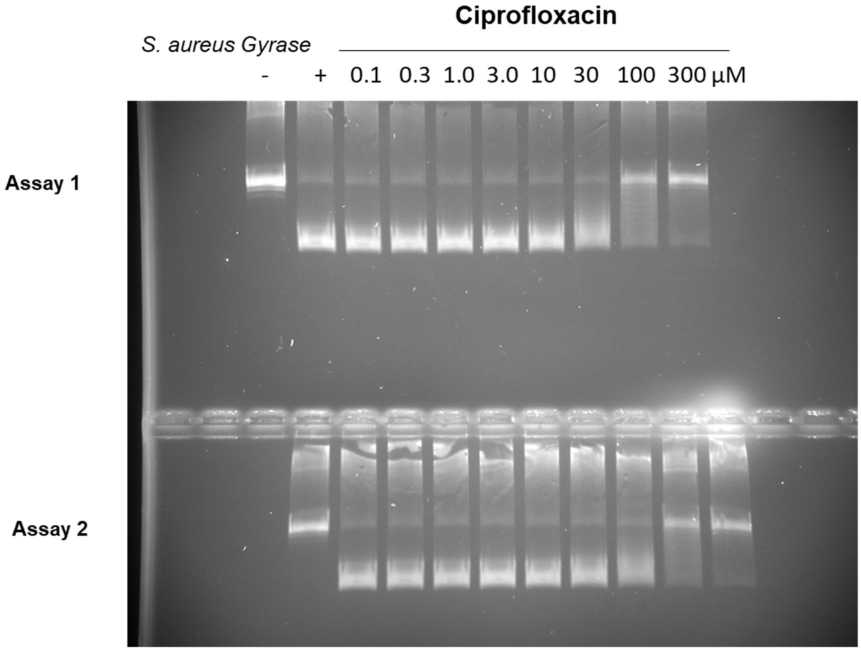

	Assay 1	Assay 2	Average IC_{50} (µM)	Average SD
ciprofloxacin	22.58	22.67	22.62	0.06

***S. aureus* Topoisomerase IV decatenation**

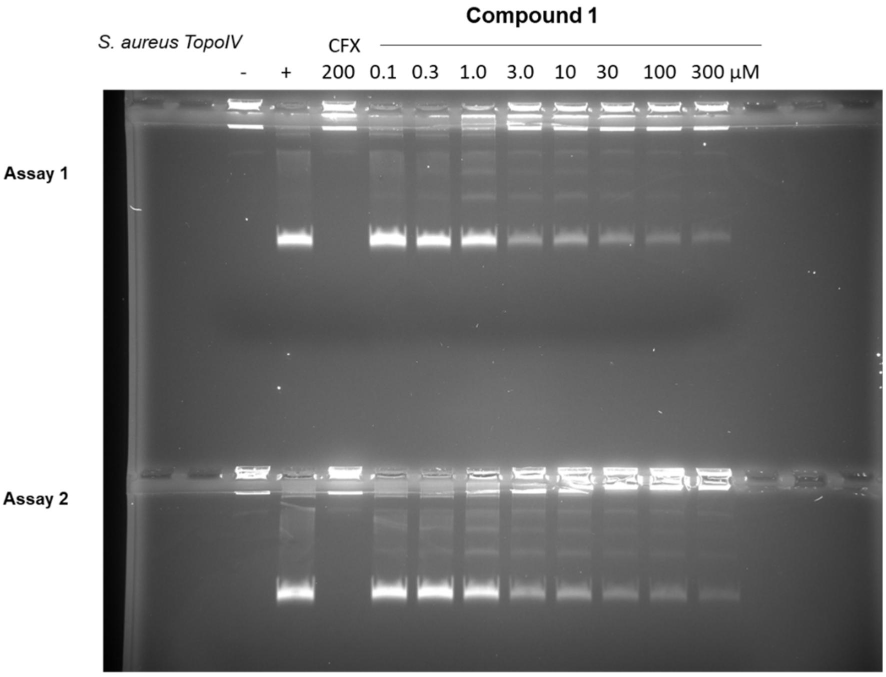

CFX= ciprofloxacin

	Assay 1	Assay 2	Average IC_{50} (μM)	Average SD
compound 1	2.2	2.3	2.25	0.07

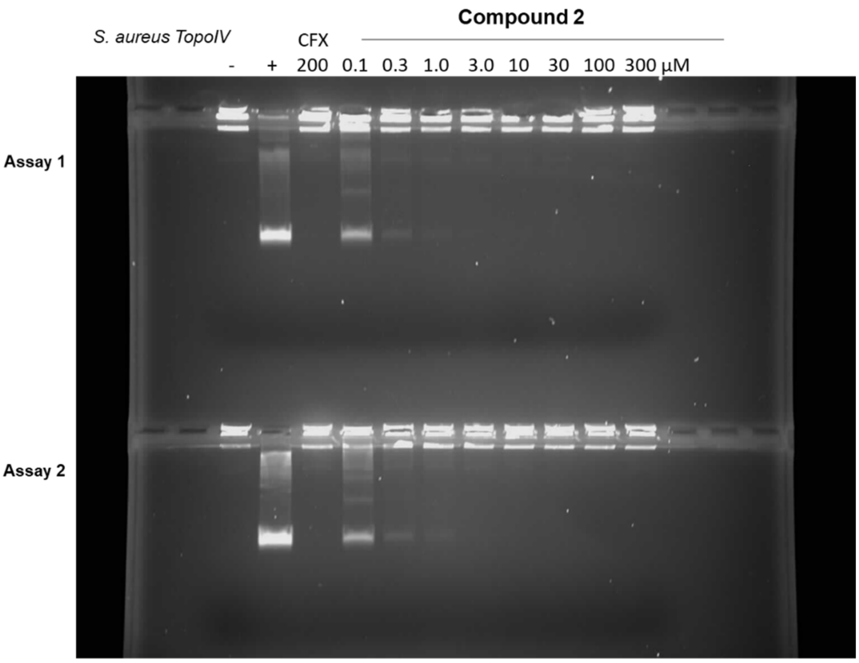

CFX= ciprofloxacin

	Assay 1	Assay 2	Average IC_{50} (μM)	Average SD
compound 2	<0.1	<0.1	<0.1	<0.1

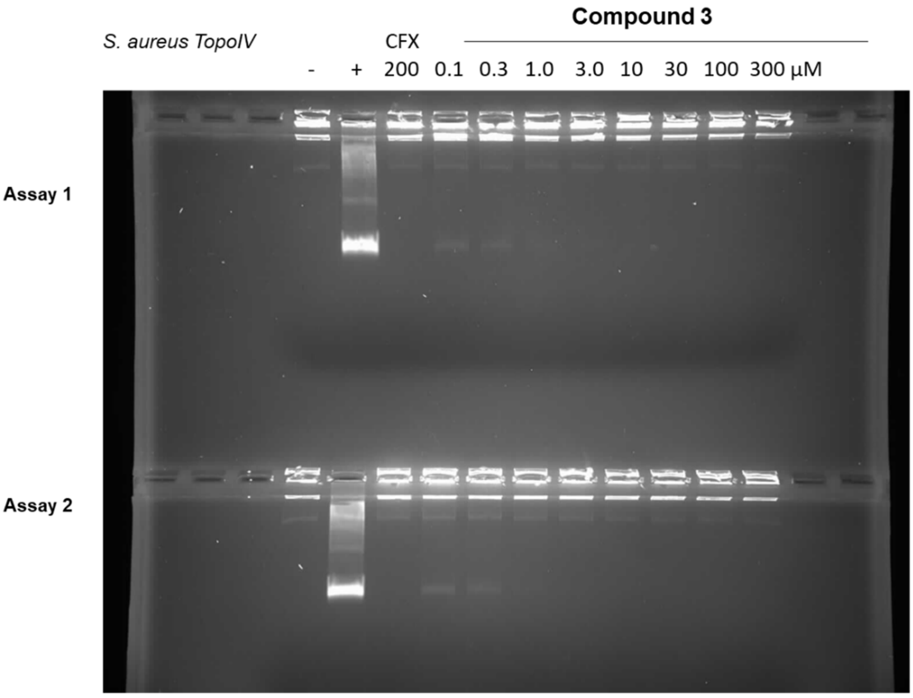

CFX= ciprofloxacin

	Assay 1	Assay 2	Average IC_{50} (µM)	Average SD
compound 3	<0.1	<0.1	<0.1	<0.1

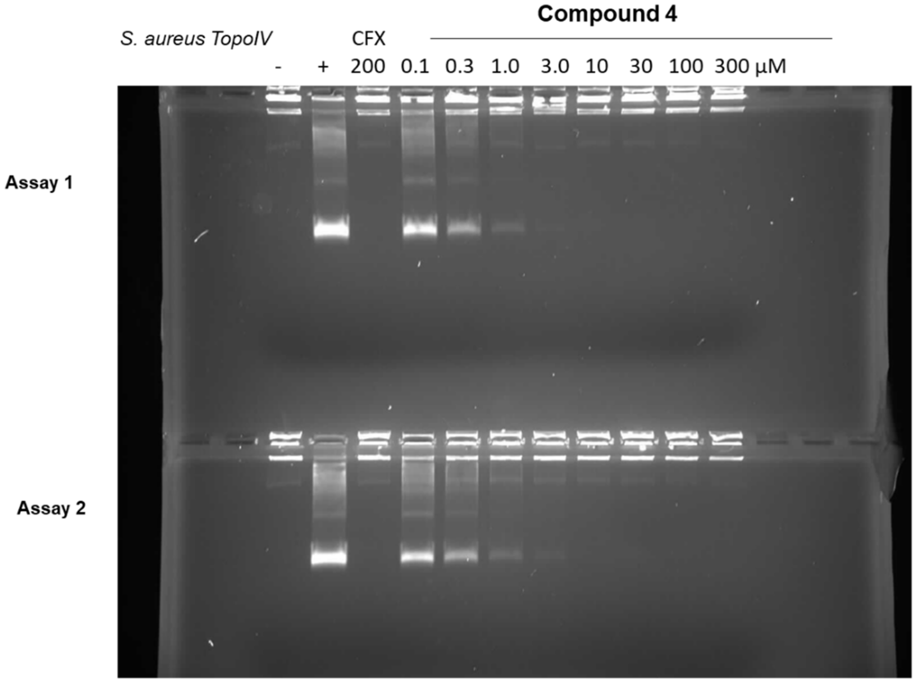

CFX= ciprofloxacin

	Assay 1	Assay 2	Average IC_{50} (µM)	Average SD
compound 4	0.26	0.17	0.22	0.06

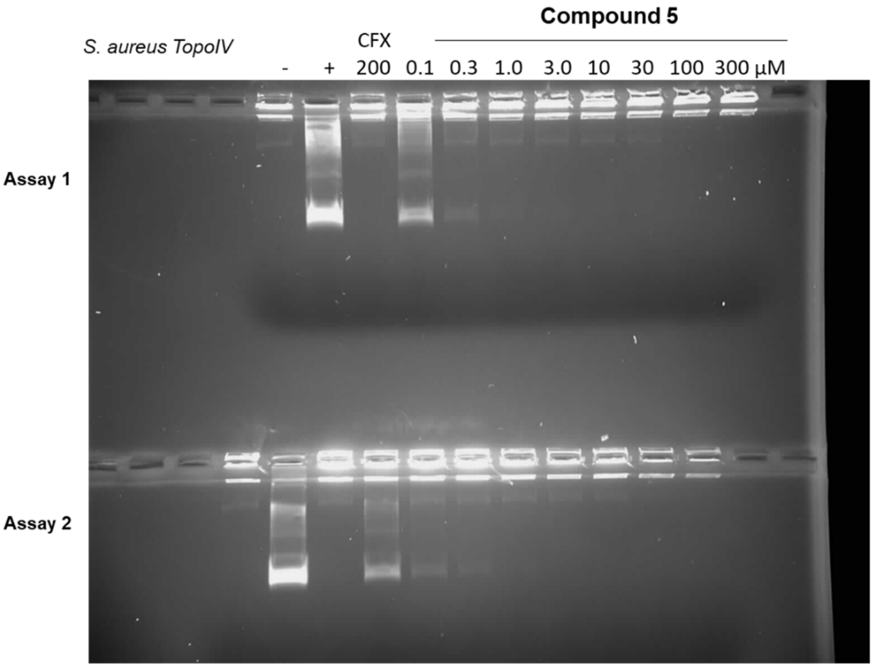

CFX= ciprofloxacin

	Assay 1	Assay 2	Average IC$_{50}$ (µM)	Average SD
compound 5	0.08	0.06	0.07	0.014

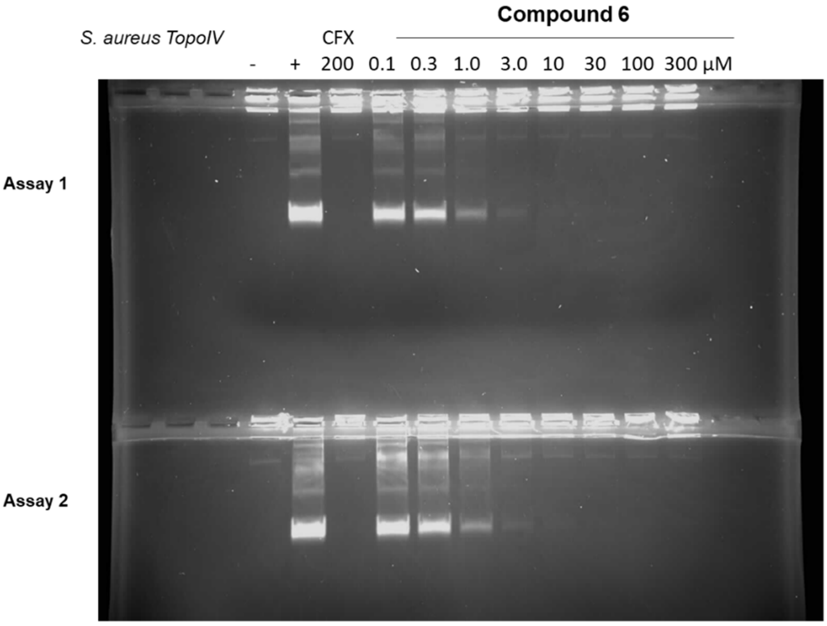

CFX= ciprofloxacin

	Assay 1	Assay 2	Average IC$_{50}$ (µM)	Average SD
compound 6	0.31	0.26	0.29	0.04

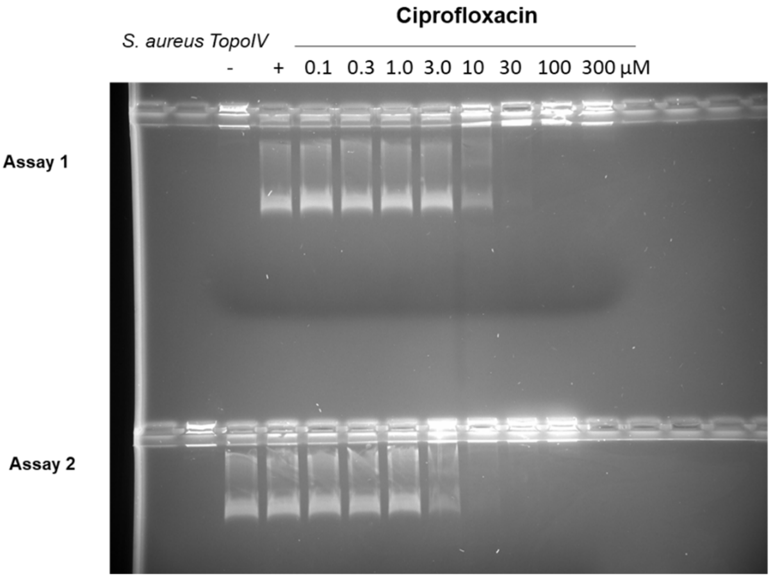

	Assay 1	Assay 2	Average IC_{50} (µM)	Average SD
ciprofloxacin	6.36	6.88	6.62	0.4

Human Topoisomerase II (TopoII) decatenation

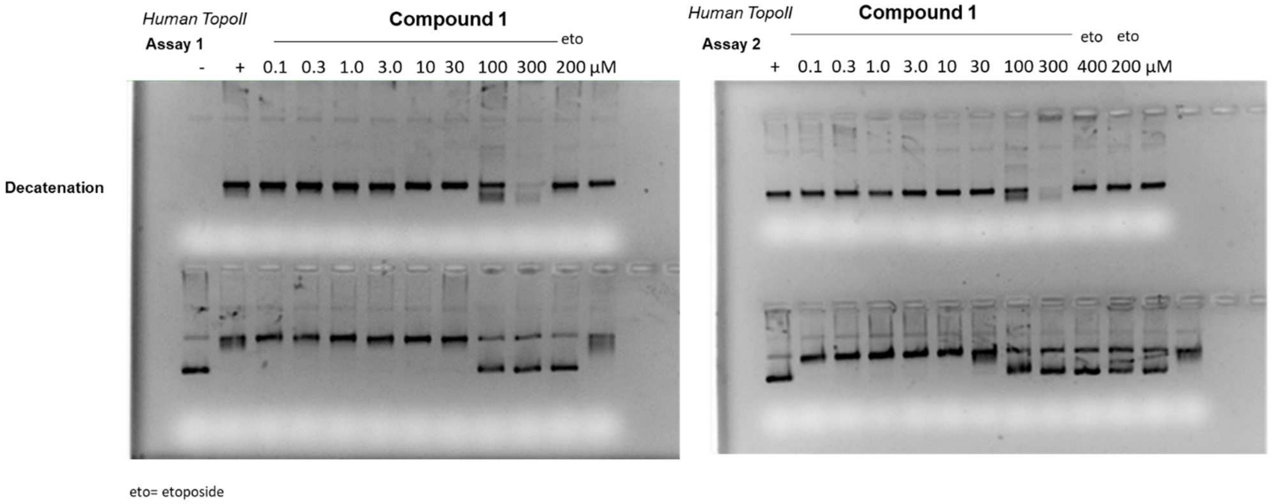

	Assay 1	Assay 2	Average IC_{50} (µM)	Average SD
Compound 1	96	99	98	2

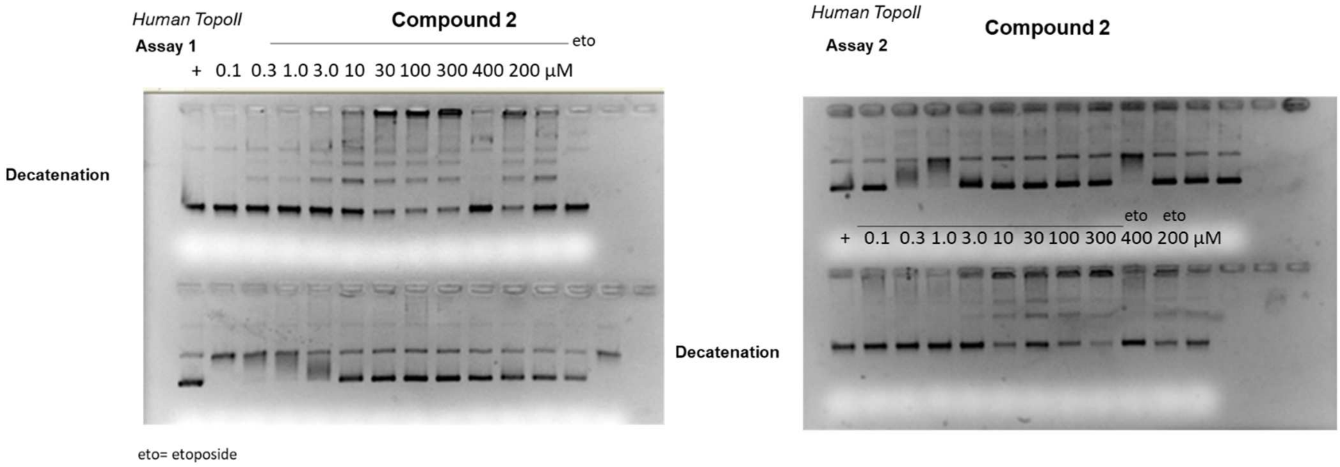

	Assay 1	Assay 2	Average IC_{50} (µM)	Average SD
Compound 2	6	7	6	0.7

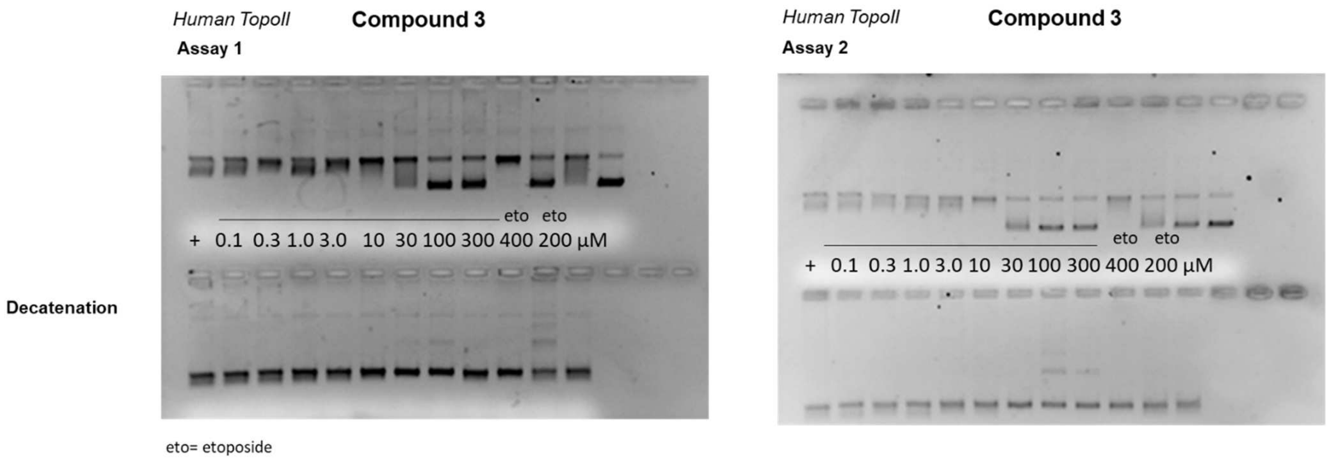

	Assay 1	Assay 2	Average IC_{50} (µM)	Average SD
Compound 3	46	66	56	14

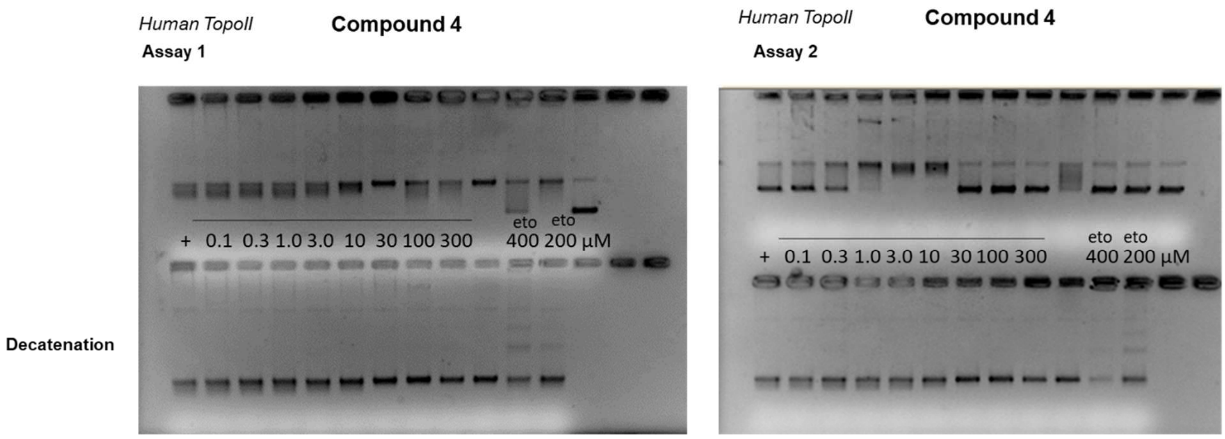

eto= etoposide

	Assay 1	Assay 2	Average IC_{50} (µM)	Average SD
Compound 4	>300	>300	>300	nd

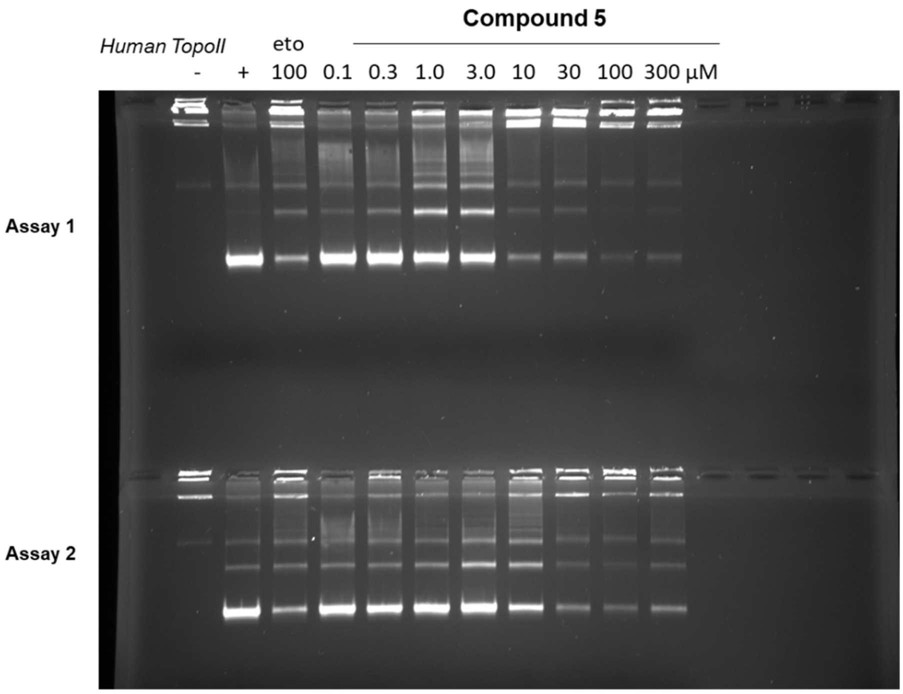

eto= etoposide

	Assay 1	Assay 2	Average IC_{50} (µM)	Average SD
Compound 5	5.45	12.8	9.12	5

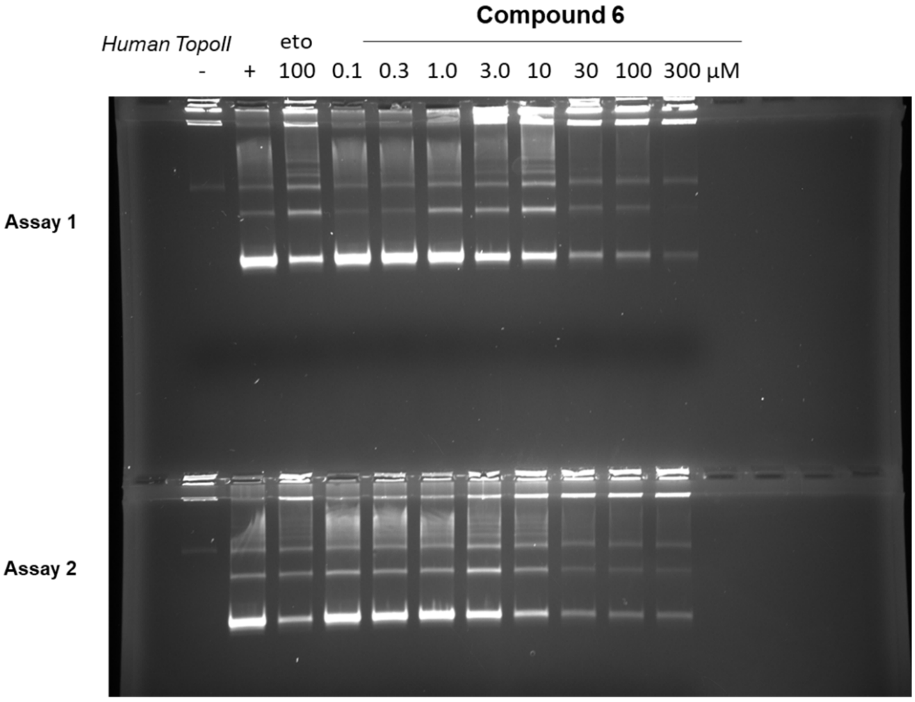

eto= etoposide

	Assay 1	Assay 2	Average IC_{50} (µM)	Average SD
Compound 6	13	7.3	10.15	4

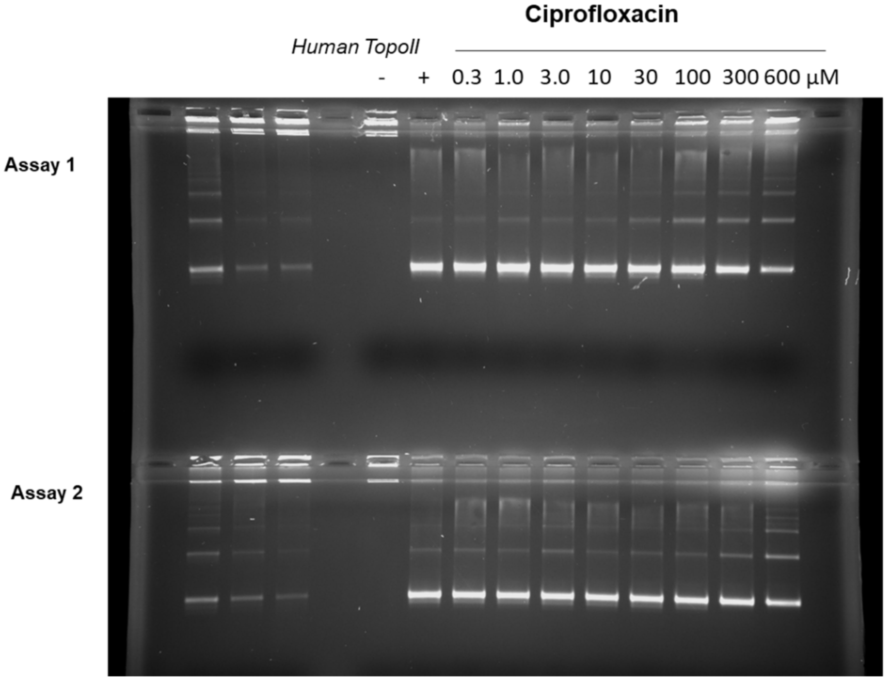

	Assay 1	Assay 2	Average IC_{50} (µM)	Average SD
ciprofloxacin	>300	>300	>300	nd
