# Supplementary material for: Antibacterial activity of novel dual bacterial DNA type II topoisomerase inhibitors
Source: PLoS One. 2020 Feb 19;15(2):e0228509. doi: 10.1371/journal.pone.0228509 (PMC7029851; doi:10.1371/journal.pone.0228509)
Supplement: S3 File — (PDF) [file pone.0228509.s003.pdf]

Growth rate of the wild type *S. aureus* ATCC BAA-1720 and of two resistant mutants

Method

To evaluate the fitness cost associated with the resistance to NBTIs, growth curves of wild type and resistant mutant strains were carried out as described in literature (Hurdle et al. JAC 2004 53:102–104). Generation times in Brain Heart Infusion broth were determined from absorbance readings taken at 675 nm. Absorbance was recorded in a microplate reader (Biotek, Winooski, VT, USA) over 24 hours. Incubation at 37°C was performed with automated shaking periods of 30s every hour, and 5s before each absorbance reading. For each strain a minimum of three independent cultures were considered to calculate the OD mean values and to determination of standard deviation.

Results

The resistance to the selected NBTIs (compound 1 and 2) in *S. aureus* ATCC BAA-1720 was associated with a fitness cost as demonstrated by the reduced bacterial growth rate of the mutants (55% and 30% reduction in mut 01 and mut 02 respectively).

Both mutants grew slower than the susceptible parent, with a generation time of 270 ± 0.1 min and 170 ± 0.1 min for mutant 01 and mutant 02 respectively, compared to a generation time of 120 ± 0.1 min for wild type strain.

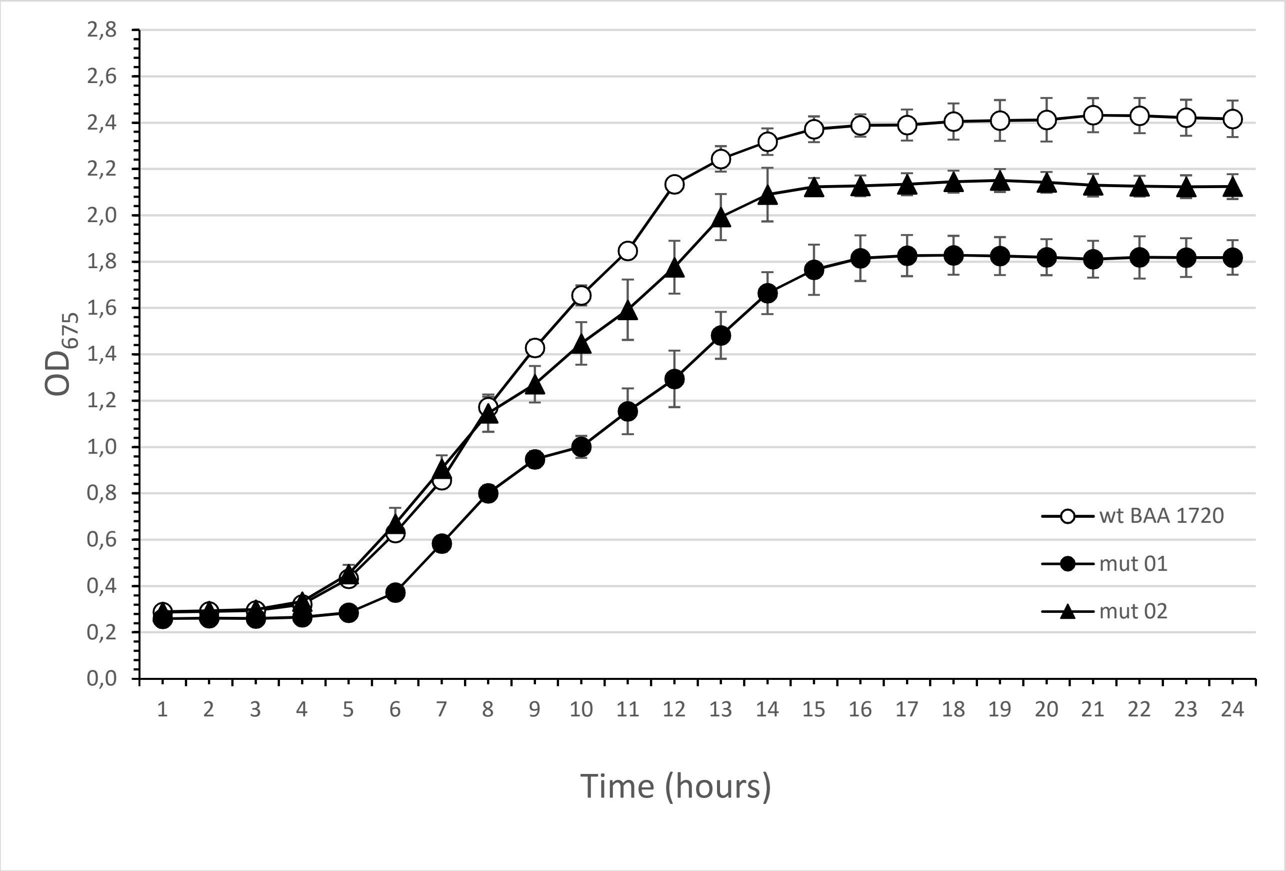

Figure 1. Growth rate of the wild type *S. aureus* ATCC BAA-1720 and of two resistant mutants. (○) *S. aureus* ATCC BAA-1720; (●) mutant strain resistant to compound 1; (▲) mutant strain resistant to compound 2. The data are shown as means±SD.
