# Supplementary material for: Antibacterial activity of novel dual bacterial DNA type II topoisomerase inhibitors
Source: PLoS One. 2020 Feb 19;15(2):e0228509. doi: 10.1371/journal.pone.0228509 (PMC7029851; doi:10.1371/journal.pone.0228509)
Supplement: S4 File — (PDF) [file pone.0228509.s004.pdf]

Susceptibility data of compounds 1 and 2 against a collection of *S. aureus* clinical isolates

compounds	*S. aureus* ATCC29213	*S. aureus* APV00045	*S. aureus* APV00280	*S. aureus* APV00281	*S. aureus* APV0048	*S. aureus* APV0049	*S. aureus* APV0052	*S. aureus* APV0053	*S. aureus* APV0063	*S. aureus* APV0064	*S. aureus* APV0065	*S.aureus*	
	ref strain	M	M	M	MLSi	MLSi	MLSc	MLSc	MRSA	MRSA	MRSA	MIC_{50} [µg/ml]	MIC_{90} [µg/ml]
1	0.25	0.5	0.12	0.12	0.25	0.5	0.5	0.25	0.12	0.12	0.12	0.125	0.5
2	≤0.06	≤0.06	≤0.06	≤0.06	≤0.06	≤0.06	≤0.06	≤0.06	≤0.06	≤0.06	≤0.06	≤0.06	≤0.06
ciprofloxacin	0.25	0.25	8	8	>8	>8	8	>8	>8	8	>8	8	8

M= macrolide resistance

iMLS= inducibile macrolide-lincosamide-streptogramin resistance

cMLS = costitutive macrolide-lincosamide-streptogramin resistance

MRSA= Methicillin-Resistant Staphylococcus Aureus
